# Role of migratory birds as a risk factor for the transmission of multidrug resistant *Salmonella enterica* and *Escherichia coli* to broiler poultry farms and its surrounding environment

**DOI:** 10.1186/s13104-024-06958-7

**Published:** 2024-10-17

**Authors:** Maram M. Tawakol, Nehal M. Nabil, Abdelhafez Samir, Heba M. Hassan, Reem M. Reda, Ola Abdelaziz, Sahar Hagag, Mona M. Elsayed

**Affiliations:** 1 Reference Laboratory for Veterinary Quality Control on Poultry Production, Animal Health Research Institute (AHRI), Agricultural Research Center (ARC), Nadi El-Seid Street, Dokki, Giza 12618 Egypt; 2 Animal Health Research Institute (AHRI), Agricultural Research Center (ARC), Hurghada, Egypt; 3 Department of Hygiene and Zoonoses, Faculty of Veterinary Medicine, Mansoura University, Mansoura, 35516 Egypt

**Keywords:** *Escherichia coli*, Migratory birds, Multidrug resistance, Poultry farms, *Salmonella*

## Abstract

Multidrug resistance (MDR) considered as global crisis facing poultry industry. Migratory birds play very important role in the dissemination of antimicrobial resistant pathogen during their fly way specially to poultry farms. Therefore, 750 samples from migratory birds and 300 samples from broiler chicken farms and its environment were collected during the winter seasons of five years (2019 to 2023). The samples were subjected to the isolation of *Salmonella enterica* and *Escherichia coli* with the detection of antimicrobial resistance (phenotypic and genotypic) with insight to the genetic similarity between the isolates from migratory birds and broiler chickens’ farms. Different members of *Enterobacteriaceae* were isolated; *Salmonella enterica*, *Escherichia coli*,* Citrobacter*, *Enterobacter*, *Klebsiella*, *Proteus*, *Providencia*, *Serratia*, *Hafnia.* 298 (28.4%) of S. enterica strains belonging to 27 serovars. *S.* Typhimurium, *S*. Kentucky, *S*. Enteritidis and *S*. Shangani were the common 4 serotypes between migratory birds and farms. Meanwhile, we found 489 (46.6%) isolates of *E. coli* belonging to 24 serogroups and O91, O128, O26, O125, O55, O103 and O159 were the common 7serogroups between migratory birds and farms samples. The majority of *Salmonella* (91.6%; 274 out of 298) and *E. coli* (92%; 450 out of 489) were MDR. The MDRI range of *Salmonella* and E. *coli* was 0.08- 1.The genetic similarity between the isolates of migratory birds and broiler chicken farms were detected by ERICPCR and hot map. This study suggests the continuous applications of surveillance programs for migratory birds and biosecurity measures in poultry farms.

**Supplementary Information:**

The online version contains supplementary material available at 10.1186/s13104-024-06958-7.

## Introduction

In the Middle East, Northern Africa, and the Mediterranean basin, Egypt considered as one of the higher population densities [1]. Bird migration is one of nature’s great mysteries and spectacles, and Egypt and the Middle East are in the epicenter of one of the world’s major migration pathways [2]. Migratory birds have the ability to spread *Salmonella* to human via shared environment, direct contact and fecal shedding [3], as well as to domestic poultry [4], resulting in both animal and human illnesses as well as significant economic losses to the poultry industry [5]. During the bird’s migration, significant impacts on the ecology and dissemination of potentially pathogenic antimicrobial resistant bacteria, including ESBL-producing *E. coli* [6] and MDR non-typhoidal *Salmonella* [7] have been arise.

Salmonellosis is one of the most important problems facing poultry industry [8–10] which results in dehydration, diarrhea, body weight loss, arthritis, pneumonia and omphalitis in the infected birds [11]. This clinical symptom is provoked by *Salmonella Gallinarum* and *Pullorum* and the birds can harbor asymptomatic Nontyphoidal Salmonella so, they are considered as silent reservoirs [12].

Also, the majority of *E. coli* produces beneficial effects such as protecting against other harmful bacteria, but when it acquires genetic material from other organisms, it converts into pathogenic [13]. The main cause of colibacillosis in poultry is avian pathogenic *E. coli* [14], it results in mortality rates of up to 30% [11], which is a significant issue for the Egyptian poultry sector [15].

Antimicrobial resistance is a serious health concern worldwide [16]. Antimicrobial resistance may be attributed to frequently use of antimicrobial drugs for therapy or as growth promoters, and poultry remains a significant source of zoonotic MDR bacteria [17]. This study was aimed to determine the role of migratory birds as a risk factor in the transmission of *Salmonella* and *E. coli* to broiler farms over a period of 5 years in three Egyptian Governorates in addition to conducting examinations on the antimicrobial susceptibility and genetic diversity of *Salmonella* and *E. coli* isolates.

## Materials and methods

### Samples collection

This study targeted 3 Egyptian Governorates in the northern delta of Egypt (Dakahlia, Damietta and Port Said). It began from the year 2019 and continued for 4 consecutive years until 2023. The sampling was conducted from October to February during each year. Thousand and fifty samples were collected including 750 sample from migratory birds (150 birds each year including 50 birds from each Governorate) that was found near to broiler farms and that sold in live bird markets in addition to 300 samples from broiler chicken and its surrounding environment. The collected migratory birds marked and photographed in order to find out the English and scientific names. The samples were included cloacal swabs from migratory birds in addition to swabs from cages and surrounding environment in the bird markets which were collected by using sterile cotton swabs pre-moisten with Buffered Peptone Water (2 ml) according to the methods described by ISO 6579-1 [18].

On the level of broiler chicken farms, the samples were collected from 300 diseased broiler chicken aged 25–40 days (60 farms each year; 20 farms from each Governorate). Clinical examinations and postmortem (PM) lesions were recorded in the investigated farms. From each farm, 10 diseased chickens were selected, sacrificed and subjected to PM examinations under septic conditions whereas samples from internal organs (liver, spleen, cecum, heart and lung) were aseptically collected by using a scalpel to cut a piece of tissue (approximately 4 cm^3^) and by sterile forceps the tissue was placed into a sterile container. The tissues were pooled together and mixed with 20 mL of BPW by stomaching for 30 s for each bird individually [19].

### Bacteriological examinations

The collected samples were subjected the isolation and identification of *Salmonella* and *E. coli*. *Salmonella* was isolated on Xylose Lysine desoxycholate (XLD) agar plates and biochemically identified according to ISO 6579 [18]. The confirmed *Salmonella* isolates were subjected to serological identification according to Kauffman – White scheme [20] to determine Somatic (O) and flagellar (H) antigens using *Salmonella* antiserum (DENKA SEIKEN Co., Japan).

*E. coli* was isolated on Eosin Methylene blue agar plates and biochemically identified according to Lee and Nolan [21]. The confirmed *E. coli* biochemically was serologically identified according to Quinnet al. [22]. by using rapid diagnostic *E. coli* antisera sets (DENKA SEIKEN Co., Japan).

### Antimicrobial susceptibility testing

The in vitro antimicrobial susceptibility of the confirmed *Salmonella* and *E. coli* was detected using the disc diffusion method on Mueller–Hinton agar (Oxoid, UK) according to the guidelines stipulated by Clinical Laboratory Standards [23] whereas the tested antimicrobial discs were categorized into sensitive, intermediate and resistant. The basis of the antimicrobial agent selection was owed to their frequent use in poultry farms in addition to their importance for human and veterinary fields. Twelve discs belonging to 8 classes were selected; quinolones (nalidixic acid; NA − 30 µg, ciprofloxacin; CP- 5 µg and levofloxacin; L- 5 µg), tetracycline (tetracycline; - T 30 µg), penicillin (ampicillin; AM- 10 µg), sulfonamides (sulfamethoxazol; SXT- 25 µg), lincosamides (clindamycin; CL- 10 µg), aminoglycosides (gentamicin; G- 10 µg, amikacin; AK- 30 µg and kanamycin; K- 30 µg), cephalosporin (cefotaxime; CF- 30 µg) and macrolides (erythromycin; E- 15 µg).

*Salmonella* Typhimurium ATCC14028 and *E. coli* ATCC 25,922 were used as control strains. MDR strains of the isolated *Salmonella* and *E. coli* were represented to exhibit resistance to three or more different antimicrobial classes [24]. Additionally, MARI was calculated using the formula (Number of antimicrobials showed resistance in each isolate/ Total number of the tested antimicrobial agents).

### Molecular detection of antimicrobial resistant genes

Based on the results of the antimicrobial susceptibility testing, conventional polymerase chain reaction technique (PCR) was used to determine the resistance genes of the most five antimicrobial agents (sulfamethoxazol, tetracycline, erythromycin, ampicillin and nalidixic acid) showed resistance in the isolated *Salmonella* and *E. coli* from both migratory birds and broiler farms. DNA was extracted from the selected isolates and examined for the presence of (sulfamethoxazol; *Sul1*, tetracycline; *Tet*A (A), erythromycin; *ere*A, ampicillin; *bla*^TEM^ and nalidixic acid; qnrA). The oligonucleotide primers that used were supplied from Metabion (Germany) (Supplementary Table 1). Agarose gel (1.5%) with ethidium bromide (5 µL) (Invitrogen, UltraPure, Waltham, MA) was prepared using Tris − acetate − EDTA buffer (0.5 M). DNA ladder (100-bp; Promega) was pipetted into the first well of each gel, with the samples loaded in the other wells of the gel (8 µL), and gel electrophoresis was performed at 135 V for 20 min. Then, the bands were detected under UV light and were photographed using gel documentation system (Alpha Innotech, Biometra) [30].

### Detection of genetic diversity by ERIC‑PCR

Four common *Salmonella* serotypes (*S*. Enteritidis, *S*. Kentucky, *S*. Typhimurium and *S*. Shangani) and 7 common *E. coli* serogroups (O26, O55, O91, O103, O128, O125 and O159) were selected and examined by ERICPCR technique to determine the similarity between the same serotypes. The extraction process was performed using QIAamp DNA mini kit (Qiagen- Germany- GmbH). The oligonucleotide primers that used were supplied from Metabion (Germany) (Supplementary Table 1). The gel was prepared and photographed as mentioned previously and the data was analyzed by the computer software.

ERIC fingerprinting data were transformed into a binary code depending on the presence or absence of each band. Dendrograms were generated by the unweighted pair group method with arithmetic average (UPGMA) and Ward’s hierarchical clustering routine. Cluster analysis and dendrogram construction were performed with SPSS, version 22 (IBM 2013) [32]. Similarity index (Jaccard / Tanimoto Coefficient and number of intersecting elements) was calculated using the online tool (https://planetcalc.com/1664/).

### Statistical analysis

Microsoft Excel (Version 15.0) was used for the data recording and SPSS (Statistical Package for Social Science) (version 25) was used to perform data analysis.

## Results

Overall, we isolated 298 (28.4%) of S. enterica strains belonging to 27 serovars from both migratory birds and poultry farms, including S. Typhimurium (*n* = 88), S. Enteritidis (*n* = 82), S. Kentucky (*n* = 45), S. Papuana (*n* = 3), S. Larochelle (*n* = 4), S. Alfort (*n* = 5), S. Shangani (*n* = 15), S. Tsevie (*n* = 7), S. Shubra (*n* = 4), S. Paratyphi A (*n* = 2), S. Heidelbergand (*n* = 8), S. Colindale (*n* = 1), S. Daula (*n* = 3), S. Bargny (*n* = 1), S. Infantis (*n* = 3), S, Inganda (*n* = 4), S. Angers (*n* = 1), S. Molade (*n* = 1), S. Newport (*n* = 3), S. Apeyeme (*n* = 2),S. Lexington (*n* = 1), S. Labadi (*n* = 1), S. Rechovot (*n* = 1), S. Tamale (*n* = 2), S. Virchow (*n* = 3), S. Wingrove (*n* = 2) and S. Montevideo (*n* = 6). There were 4 common serovars (S. Typhimurium, S. Enteritidis, S. Kentucky and S. Shangani) between migratory birds and poultry farms isolates. Regarding to the *E. coli*, we isolated 489 (46.6%) isolates of *E. coli* belonging to 24 serogroups, including O1 (n14), O2 (*n* = 8), O103 (*n* = 7), O124 (*n* = 7), O125 (*n* = 16), O128 (*n* = 39), O142 (*n* = 6), O144 (*n* = 13), O151 (*n* = 8), O158 (*n* = 2), O159 (*n* = 4), O166 (*n* = 14), O26 (*n* = 24), O55 (*n* = 11), O28 (*n* = 9), O6 (*n* = 6), O63 (*n* = 10), O86 (*n* = 16), O91 (*n* = 39), O146 (*n* = 7), O15 (*n* = 4), O163 (*n* = 5), O17 (*n* = 5) and O78 (*n* = 40).

The recorded results in Table 1 revealed that the prevalence of *S. enterica* was 28.4% (298 isolates out of 1050 sample) including 169 isolates from migratory birds and 129 isolates from the examined poultry farms. On the level of migratory birds, the prevalence of *S. enterica* was 21.3% in 2019, 24.7% in 2020, 28% in 2021, 16.7% in 2022 and 22% in 2023. On the farm level, *Salmonella* was isolated with 46.7% in 2019, 46.7% in 2020, 33.3% in 2021, 45% in 2022 and 43.3% in 2023. Regarding to the *E. coli* prevalence during the five years of the study was 46.6% (489 out of 1050) including 244 isolates from migratory birds and 255 isolates from poultry farms. On the level of migratory birds, it was 38.7% in 2019, 26.7% in 2020, 34% in 2021, 40.7% in 2022 and 22.7% in 2023. Meanwhile, the prevalence of *E. coli* in poultry farms was 78.3% in 2019, 83.3% in 2020, 76.7% in 2021, 86.7% in 2022 and 83.3% in 2023. The higher isolation was found in the year 2022 for both migratory bird with 40.7% and for farms with (86.7%). Meanwhile, the lower isolation was recorded from migratory birds in 2023 with 22.7%and from farms in 2021 with 76.7%. Another seven species were recorded; *Citrobacter*, *Enterobacter*, *Klebsiella*, *Proteus*, *Providencia*, *Serratia*, *Hafnia* and all of them were isolated from the collected samples in the years of study except for *Hafnia* that recorded only from migratory birds in 2019. The results in Table 1 revealed that *Proteus* and *Klebsiella spp.* were the most 2 species showed the highest isolation after *Salmonella* and *E. coli* was 26.7% in 2019 and 43.3% in 2020 from migratory birds and farms, respectively.

**Table 1:** Prevalence of *Enterobacteriaceae* isolates from migratory birds and broiler chicken farms

Enterobacteriaceae Member	Migratory birds (150 birds/ year)						Broiler farms (60 farms/ year)					
	Total no. of isolates (%)	2019	2020	2021	2022	2023	Total no. of isolates (%)	2019	2020	2021	2022	2023
Salmonella spp.	169/750 (22.5)	32/150 (21.3%)	37/150 (24.7%)	42/150 (28%)	25/150 (16.7%)	33/150 (22%)	129/300 (43)	28/60 (46.7%)	28/60 (46.7%)	20/60 (33.3%)	27/60 (45%)	26/60 (43.3%)
*E. coli* spp.	244/750 (32.5)	58/150 (38.7%)	40/150 (26.7%)	51/150 (34%)	61/150 (40.7%)	34/150 (22.7%)	245/300 (81.7)	47/60 (78.3%)	50/60 (83.3%)	46/60 (76.7%)	52/60 (86.7%)	50/60 (83.3%)
*Citrobacter* spp.	147/750 (19.6)	19/150 (12.7%)	25/150 (16.7%)	35/150 (23.3%)	33/150 (22%)	35/150 (23.3%)	42/300 (14)	4/60 (6.7%)	9/60 (15%)	9/60 (15%)	8/60 (13.3%)	12/60 (20%)
*Enterobacter* spp.	110/750 (14.67)	29/150 (19.3%)	34/150 (22.7%)	18/150 (12%)	9/150 (6%)	20/150 (13.3%)	48/300 (16)	13/60 (21.7%)	5/60 (8.3%)	7/60 (11.7%)	9/60 (15%)	14/60 (23.3%)
*Klebsiella* spp.	151/750 (20.13)	18/150 (12%)	40/150 (26.7%)	35/150 (23.3%)	28/150 (18.7%)	30/150 (20%)	106/300 (35.33)	23/60 (38.3%)	26/60 (43.3%)	19/60 (31.7%)	24/60 (40%)	14/60 (23.3%)
*Proteus* spp.	143/750 (19.07)	40/150 (26.7%)	15/150 (10%)	31/150 (20.7%)	32/150 (21.3%)	25/150 (16.7%)	66/300 (22)	16/60 (26.7%)	14/60 (23.3%)	14/60 (23.3%)	13/60 (21.7%)	9/60 (15%)
*Providencia* spp.	80/750 (10.67)	9/150 (6%)	20/150 (13.3%)	10/150 (6.7%)	17/150 (11.3%)	24/150 (16%)	26/300 (8.67)	3/60 (5%)	6/60 (10%)	10/60 (16.7%)	2/60 (3.3%)	5/60 (8.3%)
*Serratia* spp.	48/750 (6.4)	7/150 (4.7%)	8/150 (5.3%)	8/150 (5.3%)	15/150 (10%)	10/150 (6.7%)	11/300 (3.67)	1/60 (1.7%)	0/60 (0%)	1/60 (1.7%)	3/60 (5%)	6/60 (10%)
*Hafnia* spp.	10/750 (1.33)	10/150 (6.7%)	0/150 (0%)	0/150 (0%)	0/150 (0%)	0/150 (0%)	0	0/60 (0%)	0/60 (0%)	0/60 (0%)	0/60 (0%)	0/60 (0%)

Concerning to the results of the serotyping (Table, 2), the results revealed that *S.* Typhimurium, *S*. Kentucky and *S*. Enteritidis were the most predominant serotypes isolated from both migratory birds and farms. Four serotypes (*S.* Typhimurium, *S*. Kentucky, *S*. Enteritidis and *S*. Shangani) were recorded as common serotypes between migratory birds and farms. From (Table 3), 3 predominant *E. coli* serogroups (O91, O26, and O128) were reported from migratory birds and broiler chicken farms. *E. coli* O2 and O78 were isolated only from migratory birds. Seven serogroups (O91, O128, O26, O125, O55, O103 and O159) were common serogroups between migratory birds and farms samples.

**Table 2:** Prevalence of the isolated Salmonella serotypes in migratory birds and broiler chicken farms

Governorate	Year	Migratory birds			Broiler farms	
		Serotype	No.	Bird spp.	Serotype	No.
Dakahlia	2019	Kentucky	10	Northern Northern shoveler	Typhimurium	3
					Kentucky	3
					Enteritidis	2
					Newport	1
					Angers	1
Damietta	2019	Enteritidis	5	Northern Northern Shoveler	Enteritidis	3
		Papuana	3	Northern Northern Shoveler	Typhimurium	3
					Inganda	2
Port Said	2019	Enteritidis	5	Northern Northern Shoveler	Typhimurium	6
		Larochelle	4	Northern Northern Shoveler	Kentucky	4
		Typhimurium	5	Northern Northern Shoveler		
Dakahlia	2020	Alfort	5	Common Teal	Typhimurium	4
		Enteritidis	5	Garganey	Enteritidis	4
		Typhimurium	5	Moorhen	Molade	1
Damietta	2020	Enteritidis	5	Moorhen	Enteritidis	5
		Shangani	4	Garganey	Shangani	3
					Daula	2
					Tamale	2
Port Said	2020	kentucky	5	Common Teal	Typhimurium	4
		Tsevie	3	Common Teal	Newport	2
		Typhimurium	5	Moorhen	Enteritidis	1
Dakahlia	2021	Tsevie	4	Garganey	Typhimurium	5
		Typhimurium	9	Common Teal- Northern Pintail	Enteritidis	2
					Kentucky	1
Damietta	2021	Enteritidis	10	Shelduck	Enteritidis	4
		Kentucky	5	Common Teal- Northern Pintail	Kentucky	3
Port Said	2021	Heidelberg	5	Common Teal	Kentucky	3
		Kentucky	5	Common Teal- Northern Pintail	Apeyeme	2
		Shubra	4	Common Teal		
Dakahlia	2022	Heidelberg	3	Common Teal	Typhimurium	4
					Virchow	3
					Colindale	1
					Lexington	1
Damietta	2022	Enteritidis	10	Northern Northern Shoveler-Northern Pintail	Kentucky	4
					Enteritidis	3
		Paratyphi A	2	Northern Pintail	Infantis	3
Port Said	2022	Typhimurium	10	Northern Pintail- Commom Pochard	Typhimurium	5
					Bargny	1
					Inganda	2
Dakahlia	2023	Shangani	5	Common Teal	Kentucky	2
		Montevideo	5	Northern Pintail	Enteritidis	2
					Shangani	3
Damietta	2023	Montevideo	1	Common Teal	Typhimurium	3
		Typhimurium	10	Northern Pintail	Enteritidis	4
		Enteritidis	5	Northern Pintail	Wingrove	2
				Northern Northern Shoveler	Labadi	1
Port Said	2023	Typhimurium	2	Moorhen (Gallinulachloropus)	Typhimurium	5
		Enteritidis	5	Common Teal (Anas Crecca)	Enteritidis	2
					Rechovot	1
					Daula	1

**Table 3:** Prevalence of the isolated *E. Coli* serogroup in migratory birds and broiler chicken farms

Governorate	Year	Migratory birds			Broiler farms		
		Serogroup	No.	Bird spp.	Serogroup	No.	
Dakahlia	2019	O91	5	Northern Northern shoveler	O1	1	
		O124	4	common teal	O91	6	
		O26	4	Northern pintail	O151	2	
		O55	5	common teal	O159	1	
		O78	5	Northern Northern shoveler	O55	3	
					O6	2	
Damietta	2019	O128	10	common teal- northern pintail	O1	1	
		O2	5	Northern pintail	O63	2	
		O78	5	common teal	O103	3	
					O128	5	
					O159	3	
Port Said	2019	O26	5	common teal	O26	9	
		O78	10	Northern Northern shoveler- common teal	O166	4	
					O144	5	
Dakahlia	2020	O125	5	common teal	O125	4	
		O128	5	Moorhen	O86	4	
		O146	3	common teal	O128	3	
		O15	4	common teal	O144	3	
		O91	4	Garganey	O91	3	
Damietta	2020	O26	10	Northern pintail- Garganey	O26	5	
		O78	5	common teal	O151	3	
					O28	5	
					O142	1	
					O166	2	
					O1	2	
Port Said	2020	O91	4	Northern pintail	O91	6	
					O86	6	
					O166	3	
Dakahlia	2021	O111	5	common teal	O125	3	
		O125	4	northern paintail	O128	3	
		O128	5	common teal	O91	5	
		O78	5	common teal	O6	2	
		O91	4	common teal			
Damietta	2021	O128	5	Shelduck	O128	6	
		O26	3	Shelduck	O26	5	
					O63	3	
					O142	2	
Port Said	2021	O128	5	Wigeon	O91	3	
		O55	5	northern paintail	O128	7	
		O78	5	common teal	O55	3	
		O91	5	common teal	O124	2	
					O1	2	
Dakahlia	2022	O111	5	Northern pintail	O125	5	
		O128	5	Northern Northern shoveler	O128	4	
		O55	5	common teal	O91	4	
		O91	9	Northern pintail- Common pochard	O1	3	
					O28	2	
Damietta	2022	O146	4	Northern pintail	O128	6	
		O128	5	Common pochard	O124	5	
		O159	10	Northern Northern shoveler	O166	2	
		O163	5	Northern Northern shoveler	O144	3	
					O6	1	
					O91	1	
Port Said	2022	O128	5	Northern pintail	O55	5	
		O2	3	Common pochard	O91	8	
		O26	5	Common pochard	O63	1	
					O166	1	
					O86	1	
Dakahlia	2023	O91	5	Northern Northern shoveler	O125	4	
		O125	4	Common pochard	O91	3	
					O151	3	
					O28	2	
					O144	2	
					O6	1	
Damietta	2023	O17	5	common teal	O128	5	
		O128	5	Northern pintail	O142	3	
		O78	5	Northern Northern shoveler	O166	2	
					O1	4	
					O86	3	
Port Said	2023	O26	5	moorhen (Gallinulachloropus)	O103	4	
		O103	5	moorhen (Gallinulachloropus)	O26	5	
					O158	2	
					O86	2	
					O1	1	
					O63	4	

The in vitro antimicrobial susceptibility of the recovered *Salmonella* and *E. coli* against 12 antimicrobial agents belonged to 8 different classes showed resistance in the majority of the examined isolates. From Table 4, the high level of resistances in *Salmonella* recovered from migratory birds during the study period were recorded in nalidixic acid (100%), tetracycline (31 to 100%), ampicillin (46.9 to 100%), sulfamethoxazol (68.8 to 100%), clindamycin (31.3 to 100%), and erythromycin (87.5 to 100%). Also, in the recovered *Salmonella* from farm, the highest resistance was found for nalidixic acid (100%), ampicillin (92.6 to 100%), sulfamethoxazol (85.7 to 100%), clindamycin (75 to 100%), and erythromycin (92.6–100%).

**Table 4:** Antimicrobial resistant pattern of the isolated salmonellae from migratory birds and broiler chicken farms

Antimicrobial Agent	Migratory birds					Broiler farms				
	2019	2020	2021	2022	2023	2019	2020	2021	2022	2023
NA	32/32 (100%)	37/37 (100%)	42/42 (100%)	25/25 (100%)	33/33 (100%)	28/28 (100%)	28/28 (100%)	20/20 (100%)	27/27 (100%)	26/26 (100%)
CP	9/32 (28.1%)	20/37 (54.1%)	13/42 (30.9%)	17/25 (68%)	11/33 (33.3%)	24/28 (85.7%)	25/28 (89.3%)	15/20 (75%)	16/27 (59.3%)	20/26 (76.9%)
L	10/32 (31.3%)	7/37 (18.9%)	9/42 (21.4%)	5/25 (20%)	0/33 (0%)	16/28 (57.1%)	19/28 (67.9%)	10/20 (50%)	12/27 (44.4%)	19/26 (73.1%)
T	14/32 (43.8%)	29/37 (78.4%)	13/42 (31%)	25/25 (100%)	14/33 (42.4%)	27/28 (96.45)	27/28 (96.4%)	18/20 (90%)	20/27 (74.1%)	22/26 (84.6%)
AM	15/32 (46.9%)	28/37 (75.7%)	32/42 (76.2%)	25/25 (100%)	31/33 (93.9%)	28/28 (100%)	28/28 (100%)	20/20 (100%)	25/27 (92.6%)	26/26 (100%)
SXT	22/32 (68.8%)	26/37 (70.3%)	29/42 (69%)	25/25 (100%)	20/33 (60.6%)	24/28 (85.7%)	28/28 (100%)	20/20 (100%)	25/27 (92.6%)	26/26 (100%)
CL	10/32 (31.3%)	24/37 (64.9%)	15/42 (35.7%)	25/25 (100%)	13/33 (39.4%)	21/28 (75%)	27/28 (96.4%)	20/20 (100%)	20/27 (74.1%)	21/26 (80.8%)
G	6/32 (18.8%)	0/37 (0%)	1/42 (2.4%)	5/25 (20%)	0/33 (0%)	17/28 (60.7%)	2/28 (7.1%)	14/20 (70%)	18/27 (66.7%)	21/26 (80.8%)
AK	9/32 (28.1%)	14/37 (37.8%)	10/42 (23.8%)	5/25 (20%)	10/33 (30.3%)	17/28 (60.7%)	20/28 (71.4%)	9/20 (45%)	12/27 (44.4%)	14/26 (53.8%)
K	7/32 (21.9%)	12/37 (32.4%)	14/42 (33.3%)	10/25 (40%)	9/33 (27.3%)	18/28 (64.3%)	20/28 (71.4%)	10/20 (45%)	12/27 (44.4%)	15/26 (53.8%)
CF	16/32 (50%)	17/37 (45.9%)	10/42 (23.8%)	5/25 (20%)	4/33 (12.1%)	13/28 (46.4%)	19/28 (67.9%)	15/20 (75%)	13/27 (48.1%)	17/26 (65.4%)
E	28/32 (87.5%)	37/37 (100%)	39/42 (92.9%)	25/25 (100%)	31/33 (93.9%)	27/28 (96.4%)	28/28 (100%)	20/20 (100%)	25/27 (92.6%)	26/26 (100%)

From Table (5), the highest resistance in *E. coli* that isolated from migratory birds during the study period were recorded in nalidixic acid (70.6 to 100%) and clindamycin (70.7 to 91.8%), and erythromycin (98.3–100%). Meanwhile, high resistant *E. coli* that recovered from broiler farms was recorded for nalidixic acid (92 to 100%), clindamycin (78.7 to 100%), cefotaxime (85.1 to 100%) and erythromycin (94.2 to 100%).

**Table 5:** Prevalence of antimicrobial resistance of the isolated *E. Coli* from migratory birds and broiler chicken farms

Antimicrobial Agent	Migratory birds					Broiler farms				
	2019	2020	2021	2022	2023	2019	2020	2021	2022	2023
NA	58/58 (100%)	31/40 (77.5%)	36/51 (70.6%)	47/61 (77%)	25/34 (73.5%)	47/47 (100%)	50/50 (100%)	46/46 (100%)	50/52 (96.2%)	46/50 (92%)
CP	30/58 (51.7%)	9/40 (22.5%)	19/51 (37.3%)	19/61 (31.1%)	5/34 (14.7%)	35/47 (74.5%)	32/50 (64%)	30/46 (65.2%)	25/52 (48.1%)	26/50 (52%)
L	37/58 (63.8%)	10/40 (22.5%)	15/51 (29.4%)	19/61 (31.1%)	10/34 (29.4%)	32/47 (68.1%)	17/50 (34%)	22/46 (47.8%)	34/52 (65.4%)	17/50 (34%)
T	46/58 (79.3%)	30/40 (75%)	44/51 (86.3%)	47/61 (77%)	25/34 (73.5%)	42/47 (89.4%)	50/50 (100%)	46/46 (100%)	50/52 (96.2%)	46/50 (92%)
AM	48/58 (82.8%)	22/40 (55%)	27/51 (52.9%)	24/61 (39.3%)	10/34 (29.4%)	41/47 (87.2%)	41/50 (82%)	36/46 (78.3%)	34/52 (65.4%)	37/50 (74%)
SXT	46/58 (79.3%)	25/40 (62.5%)	34/51 (66.7%)	34/61 (55.7%)	19/34 (55.9%)	46/47 (97.9%)	44/50 (88%)	42/46 (91.3%)	48/52 (92.3%)	48/50 (96%)
CL	41/58 (70.7%)	29/40 (72.5%)	46/51 (90.2%)	56/61 (91.8%)	32/34 (94.1%)	37/47 (78.7%)	48/50 (96%)	46/46 (100%)	50/52 (96.2%)	45/50 (90%)
G	28/58 (48.3%)	8/40 (20%)	13/51 (25.5%)	19/61 (31.1%)	9/34 (26.5%)	27/47 (57.4%)	31/50 (62%)	28/46 (60.9%)	31/52 (59.6%)	23/50 (46%)
AK	38/58 (65.5%)	3/40 (7.5%)	7/51 (13.7%)	9/61 (14.8%)	0/34 (0%)	22/47 (46.8%)	20/50 (40%)	9/46 (19.6%)	29/52 (55.8%)	28/50 (56%)
K	30/58 (51.7%)	8/40 (20%)	14/51 (27.5%)	14/61 (23%)	0/34 (0%)	16/47 (34%)	22/50 (44%)	14/46 (30.4%)	26/52 (50%)	27/50 (54%)
CF	52/58 (89.7%)	23/40 (57.5%)	33/51 (64.7%)	34/61 (55.7%)	20/34 (58.8%)	40/47 (85.1%)	43/50 (86%)	46/46 (100%)	48/52 (92.3%)	44/50 (88%)
E	57/58 (98.3%)	40/40 (100%)	51/51 (100%)	61/61 (100%)	34/34 (100%)	45/47 (95.7%)	50/50 (100%)	46/46 (100%)	49/52 (94.2%)	49/50 (98%)

The majority of *Salmonella* (91.6%; 274 out of 298) and *E. coli* (92%; 450 out of 489) strains from both migratory birds and poultry farms showed MDR to the tested antimicrobial agents. The MDRI was reported in *Salmonella* isolated from migratory bird and farms which ranged from 0.17 to 1 and from 0.33 to 1, respectively. Regarding to *E. coli*, the MDRI in migratory birds ranged from 0.08 to 1 but in farms ranged from 0.33 to 1. On the level of farms, the different antimicrobial resistance patterns for *Salmonella* strains were 44 at which NA, E, AM, SXT, CL, T, CP was the most common one and for *E. coli* strains were 66 and E, CL, T, NA, CF, SXT, AM, CP, G, L, K, AK was the most common one (Supplementary Tables 3 &4). Meanwhile, on the level of migratory birds there were 50 different antimicrobial resistance patterns for *Salmonella* strains at which NA, E, AM, SXT was the most common pattern and 61 different patterns for *E. coli* strains at which E, CL, T, NA, CF, SXT was the most common one.

**Fig. 1:**
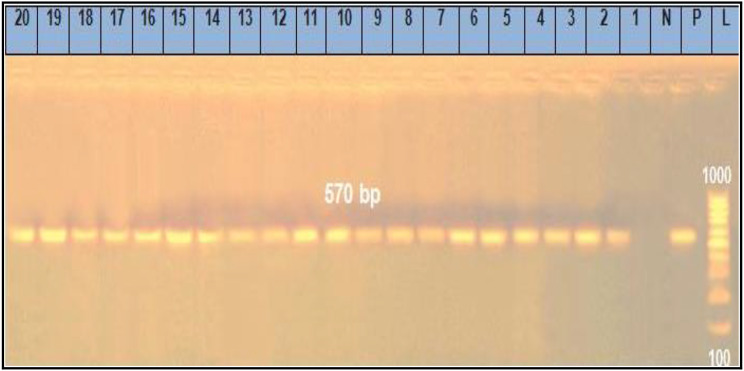
Representative full length of agarose gel electrophoresis of PCR products for *E. coli* (Lanes: 1–10) *and Salmonella* (Lanes: 11–20) isolates to detect *Tet A gene* in genomic DNA at 570 bp. Lane L: DNA ladder, P: Positive control, N: Negative control and Lanes: 2 to 5 were positive *E. coli* isolates from migratory birds in poultry farms. Lanes: 6 to 10 were positive *E. coli* isolates from migratory birds in live bird markets. Lanes: 11 to 15 were positive *Salmonella* isolates from migratory birds in poultry farms. Lanes: 16 to 20 were positive *Salmonella* isolates from migratory birds in live bird markets

**Fig. 2:**
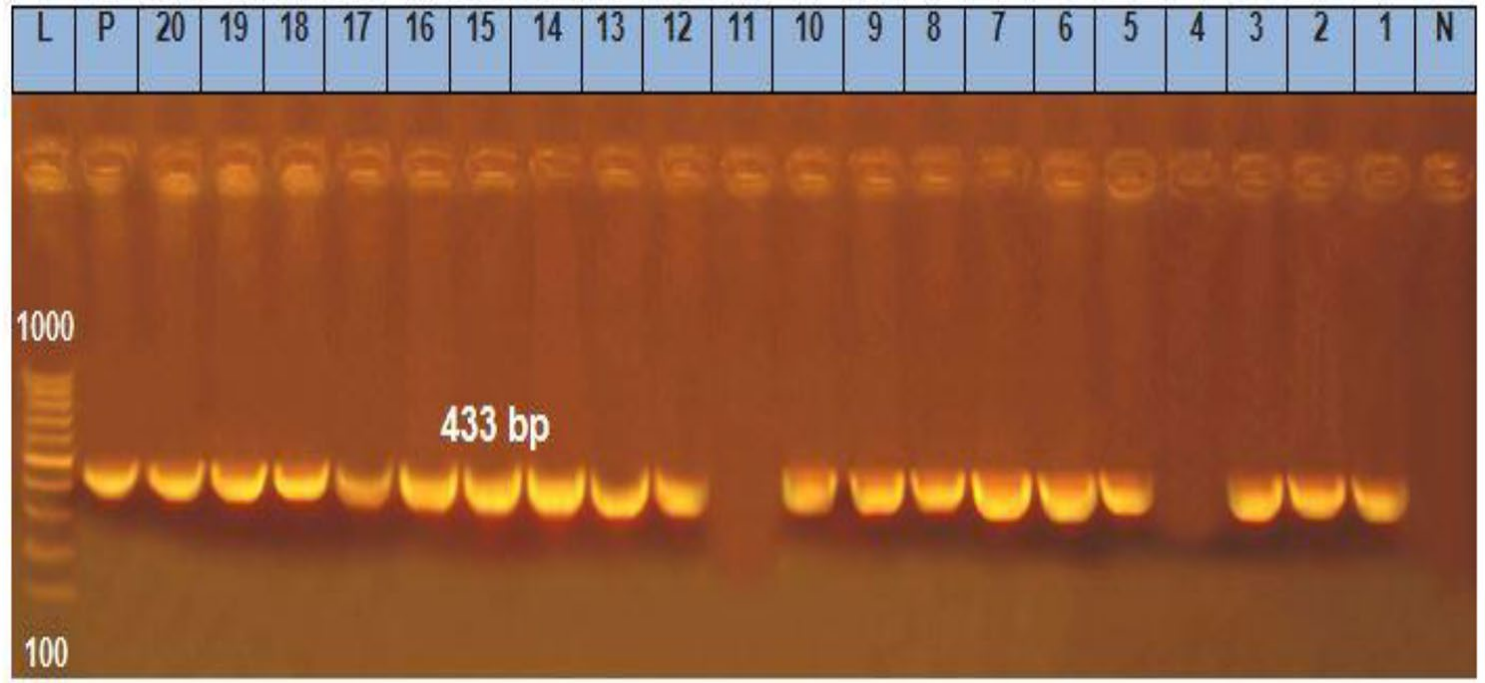
Representative full length of agarose gel electrophoresis of PCR products for *E. coli* (Lanes: 1–10) *and Salmonella* (Lanes: 11–20) isolates to detect Sul 1 *gene* in genomic DNA at 433 bp. Lane L: DNA ladder, P: Positive control, N: Negative control and Lanes: 1 to 5 except 4 were positive *E. coli* isolates from migratory birds in poultry farms. Lanes: 6 to 10 were positive *E. coli* isolates from migratory birds in live bird markets. Lanes: 12 to 15 were positive *Salmonella* isolates from migratory birds in poultry farms. Lanes: 16 to 20 were positive *Salmonella* isolates from migratory birds in live bird markets

**Fig. 3:**
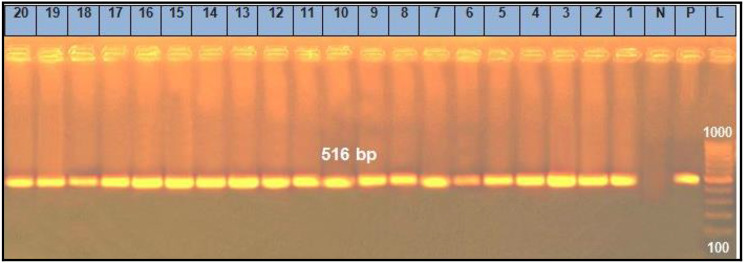
Representative full length of agarose gel electrophoresis of PCR products for *E. coli* (Lanes: 1–10) *and Salmonella* (Lanes: 11–20) isolates to detect *qnrA gene* in genomic DNA at 516 bp. Lane L: DNA ladder, P: Positive control, N: Negative control and Lanes: 1 to 5 were positive *E. coli* isolates from migratory birds in poultry farms. Lanes: 6 to 10 were positive *E. coli* isolates from migratory birds in live bird markets. Lanes: 11 to 15 were positive *Salmonella* isolates from migratory birds in poultry farms. Lanes: 16 to 20 were positive *Salmonella* isolates from migratory birds in live bird markets

**Fig. 4:**
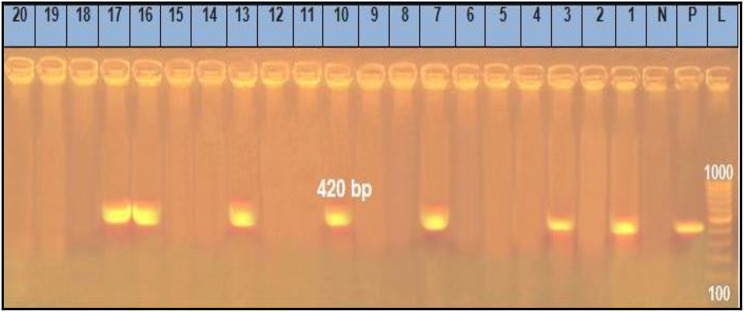
Representative full length of agarose gel electrophoresis of PCR products for *E. coli* (Lanes: 1–10) *and Salmonella* isolates (Lanes: 11–20) to detect *ereA gene* in genomic DNA at 420 bp. Lane L: DNA ladder, P: Positive control, N: Negative control and Lanes: 1 and 3 were positive *E. coli* isolates from migratory birds in poultry farms. Lanes: 7 and 10 were positive *E. coli* isolates from migratory birds in live bird markets. Lanes: 13 were positive *Salmonella* isolates from migratory birds in poultry farms. Lanes: 16 and 17 were positive *Salmonella* isolates from migratory birds in live bird markets

**Fig. 5:**
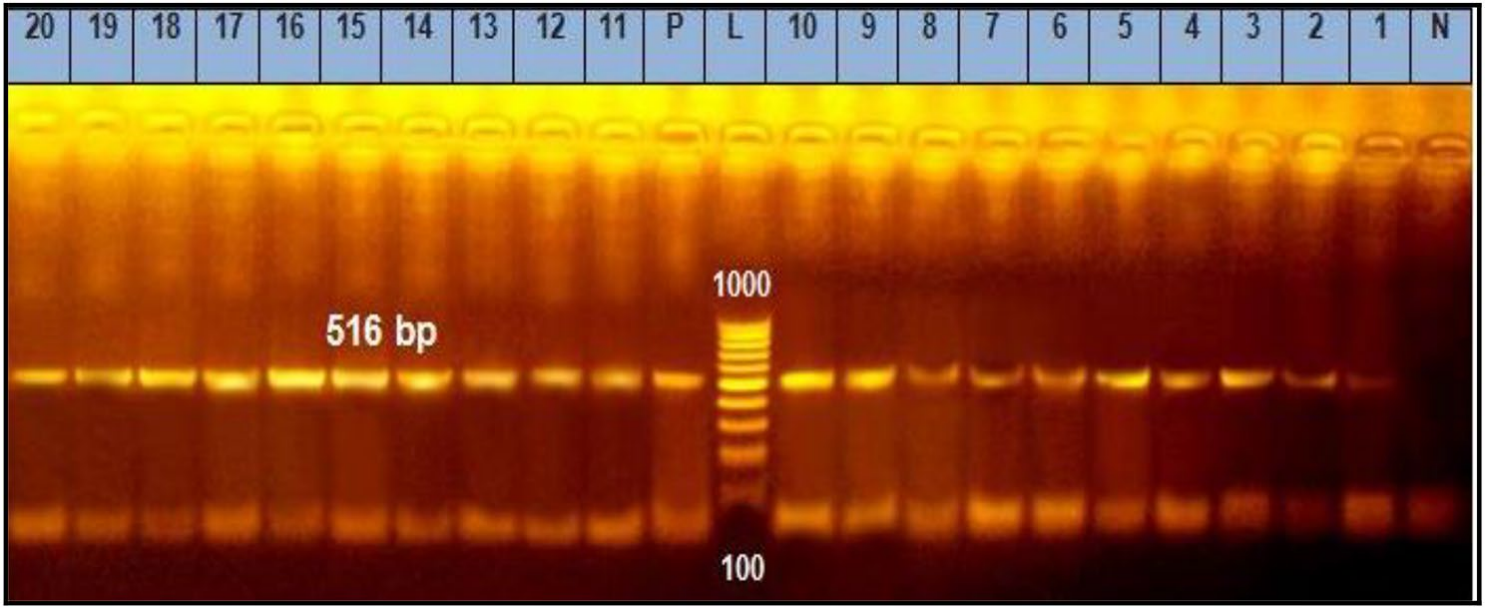
Representative full length of agarose gel electrophoresis of PCR products for *E. coli* (Lanes: 1–10) *and Salmonella* isolates (Lanes: 11–20) to detect *bla*^*TEM*^*gene* in genomic DNA at 516 bp. Lane L: DNA ladder, P: Positive control, N: Negative control and Lanes: 1 to 5 were positive *E. coli* isolates from migratory birds in poultry farms. Lanes: 6 and 10 were positive *E. coli* isolates from migratory birds in live bird markets. Lanes: 11 to 15 were positive *Salmonella* isolates from migratory birds of poultry farms. Lanes: 16 and 20 were positive *Salmonella* isolates from migratory birds of live bird markets

The recorded results in Table 6 demonstrated that the prevalence of *Sul1*, *Tet*A (A), *bla*^TEM^, *ere* A and *qnr*A genes (Figs. 1, 2, 3, 4 and 5) in the resistant *Salmonella* strains from migratory birds were 96.7%, 82%, 41.3%, 94.7% and 96.3%, respectively, but its prevalence in broiler farms were 96.7%, 95.6%, 46.8%, 97.6% and 98.4%, respectively. Meanwhile, the prevalence of *Sul1*, *Tet*A (A), *bla*^TEM^ and *qnr*A genes in the resistant *E. coli* strains which isolated from migratory birds were 95.6%, 91.7%, 36.6%, 97.7% and 98.5%, respectively, and in broiler farms were 98.7%, 96.6%, 34.7%, 97.4% and 98.3%, respectively (Figs. 1, 2, 3, 4 and 5).

**Table 6:** Prevalence and distribution of resistance genes in the isolated Salmonella strains

Agent	Isolate	Gene	Migratory birds						Broiler farms					
			Total no. of positive isolates (%)	2019	2020	2021	2022	2023	Total no. of positive isolates (%)	2019	2020	2021	2022	2023
SXT	Salmonella	*Sul*1	118/122 (96.7)	22/22 100%	25/26 96.2%	26/29 89.7%	25/25 100%	20/20 100%	119/123 (96.7)	24/24 100%	26/28 92.9%	20/20 100%	23/25 92%	26/26 100%
T		*Tet*A (A)	78/95 (82)	10/14 71.4%	20/29 69%	13/13 100%	21/25 84%	14/14 100%	109/114 (95.6)	27/27 100%	25/27 92.6%	18/18 100%	17/20 85%	22/22 100%
E		*ere*A	66/160 (41.3)	10/28 35.7%	14/37 37.8%	17/39 43.6%	15/25 60%	10/31 32.3%	59/126 (46.8)	13/27 48.1%	15/28 53.6%	7/20 35%	10/25 40%	14/26 53.9%
AM		*bla*TEM	124/131 (94.7)	15/15 100%	26/28 92.9%	30/32 93.8%	23/25 92%	30/31 96.8%	124/127 (97.6)	28/28 100%	28/28 100%	18/20 90%	24/25 96%	26/26 100%
NA		*qnr*A	163/169 (96.4)	32/32 100%	37/37 100%	42/42 100%	20/25 80%	32/33 97%	127/129 (98.4)	28/28 100%	28/28 100%	20/20 100%	25/27 92.6%	26/26 100%
SXT	*E. coli*	*Sul*1	151/158 (95.6)	43/46 93.5%	25/25 100%	31/34 91.2%	34/34 100%	18/19 94.7%	225/228 (98.7)	45/46 97.8%	44/44 100%	40/42 95.2%	48/48 100%	48/48 100%
T		*Tet*A (A)	176/192 (91.7)	46/46 100%	27/30 90%	40/44 90.9%	40/47 85.1%	23/25 92%	226/234 (96.6)	40/42 95.2%	50/50 100%	41/46 89.1%	49/50 98%	46/46 100%
E		*ere*A	89/243 (36.6)	12/57 21.1%	20/40 50%	15/51 29.4%	33/61 54.1%	9/34 26.5%	83/239 (34.7)	18/45 40%	20/50 40%	15/46 32.6%	11/49 22.4%	19/49 38.8%
AM		*bla*TEM	128/131 (97.7)	48/48 100%	22/22 100%	24/27 88.9%	24/24 100%	10/10 100%	184/189 (97.4)	41/41 100%	38/41 92.7%	35/36 97.2%	33/34 97.1%	37/37 100%
NA		*qnr*A	194/197 (98.5)	56/58 96.6%	31/31 100%	35/36 97.2%	47/47 100%	25/25 100%	235/239 (98.3)	47/47 100%	47/50 94%	46/46 100%	49/50 98%	46/46 100%

**Fig. 6:**
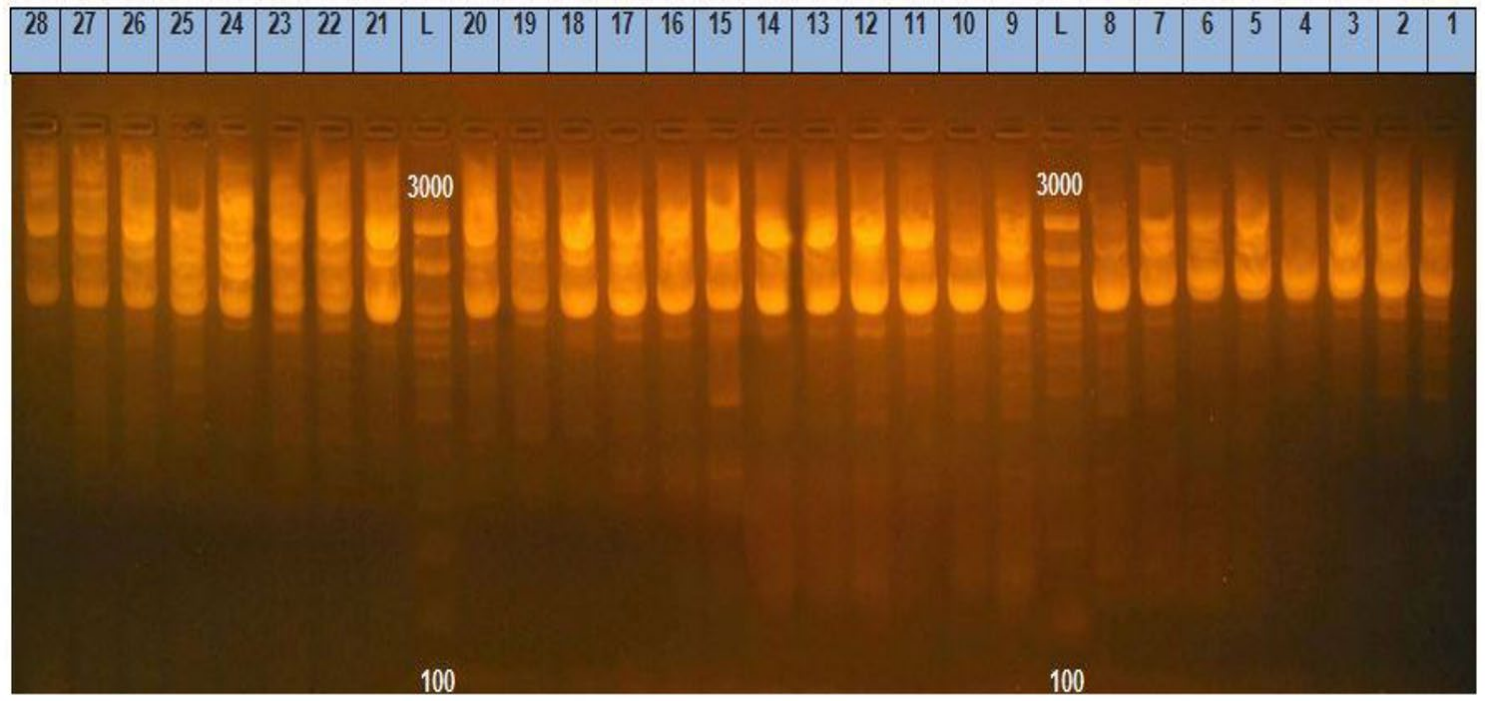
Gel electrophoresis of PCR products for 28 *E. coli* strains which produced 1–4 bands whose size ranged from 2000 to 2950 bp. Lanes; 1–10,14, 16, 23, 24, 26 &27 from farms and lanes; 11–13, 15, 17–22, 25 & 28 from migratory birds

**Fig. 7:**
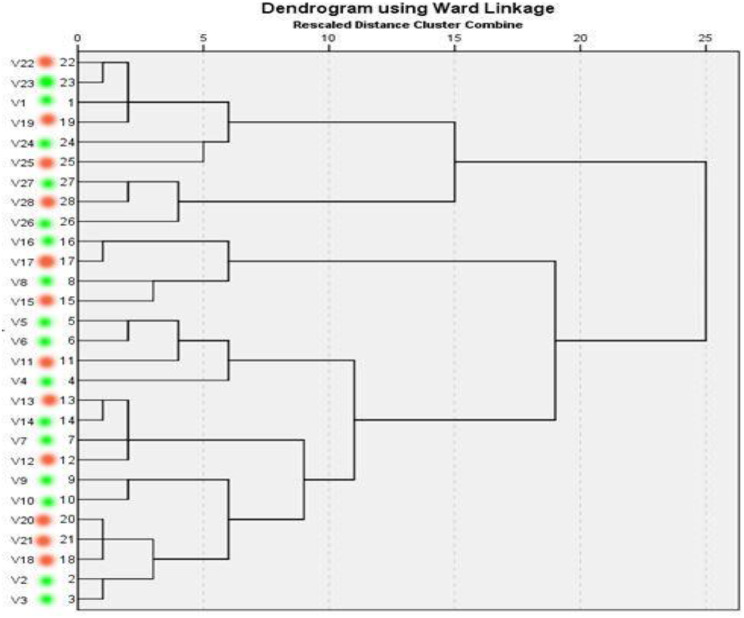
The electrophoretic profile of DNA fragments obtained from 16 *Salmonella* strains which produced 5–8 bands whose size ranged from 100 to 2974 bp. Lanes; 1,3, 4, 6, 8–12 & 16 from farms and lanes; 2, 5, 7& 13–15 from migratory birds

**Fig. 8:**
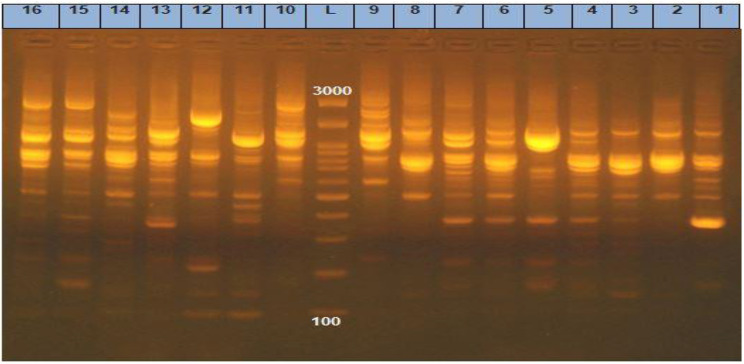
Dendrogram representing genetic relationships between *E. coli* isolates from migratory birds (isolate ID 22, 19, 25, 28, 17, 15, 11, 12,13, 18, 20 & 21; which marked with red color) and broiler chicken farms (isolates ID 23,1, 24, 27,16, 26, 8, 4, 5, 6, 7, 14, 2, 3, 9 & 10; which marked with green color) based on ERIC-PCR fingerprints. Eleven ERIC profile represented by A–J and the isolates ID represented by 1–28

**Fig. 9:**
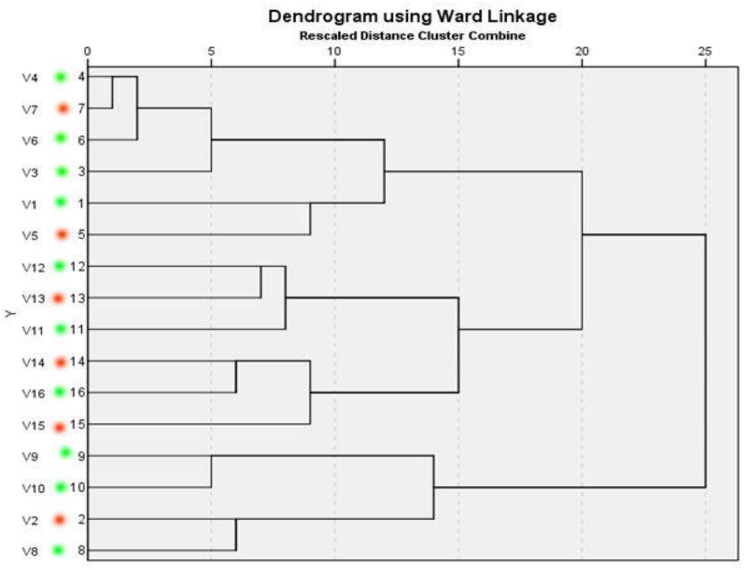
Dendrogram representing genetic relationships between Salmonella isolates from migratory birds (isolate ID 7, 5, 13, 14, 15 & 2; which marked with red color) and broiler chicken farms (isolates ID 4, 6,3,1, 12, 11, 16, 9, 10 & 8; which marked with green color) based on ERIC-PCR fingerprints. Fourteen ERIC profile represented by A–N and the isolates ID represented by 1–16

ERIC showed high genetic similarity between the bacterial strains. The electrophoretic profile of DNA fragments obtained from 28 *E. coli* strains (Figs. 6) and 16 *Salmonella* strains (Fig. 7) produced 1–4 bands for *E. coli* and 5–8 bands for *Salmonella* strains, whose size ranged from 2000 to 2950 bp for and from 100 to 2974, respectively. *Salmonella* strains were clustered into 2 groups including X1 (*n* = 12) and X2 (*n* = 4), also *E. coli* strains were clustered into 2 groups as X1 (*n* = 9) and X2 (*n* = 19). For *E. coli*, the genetic similarity was found between many isolates for example No. 22 & 19 with isolates No. 23&1 from migratory birds and broiler chicken farms, respectively (Fig. 8). There were 14 different ERIC types were found in the examined *Salmonella* isolates (Fig. 9). The similarity was found only between isolate No. 7 from migratory birds and isolates No. 4 & 6 from broiler chicken farms.

**Fig. 10:**
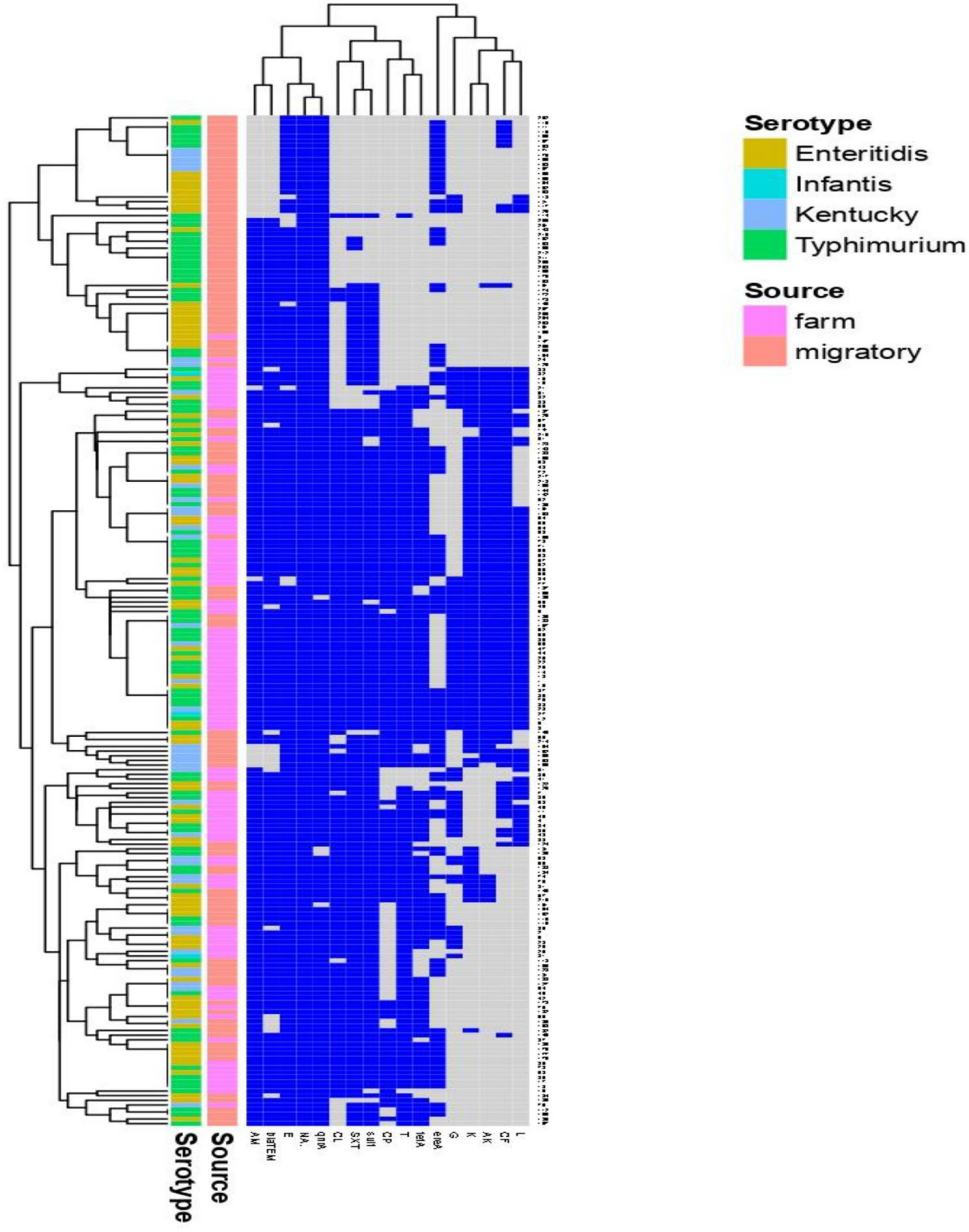
*Salmonella* Enteritidis, *S*. Typhimurum, *S*. Kentaky and *S*. Infantis (the most important Pathogenic strains) which were isolated from migratory birds and broiler farms were selected and used to construct a heatmap. Clustering demonstrated predominant multidrug resistant strains were isolated from migratory birds and broiler farms

**Fig. 11:**
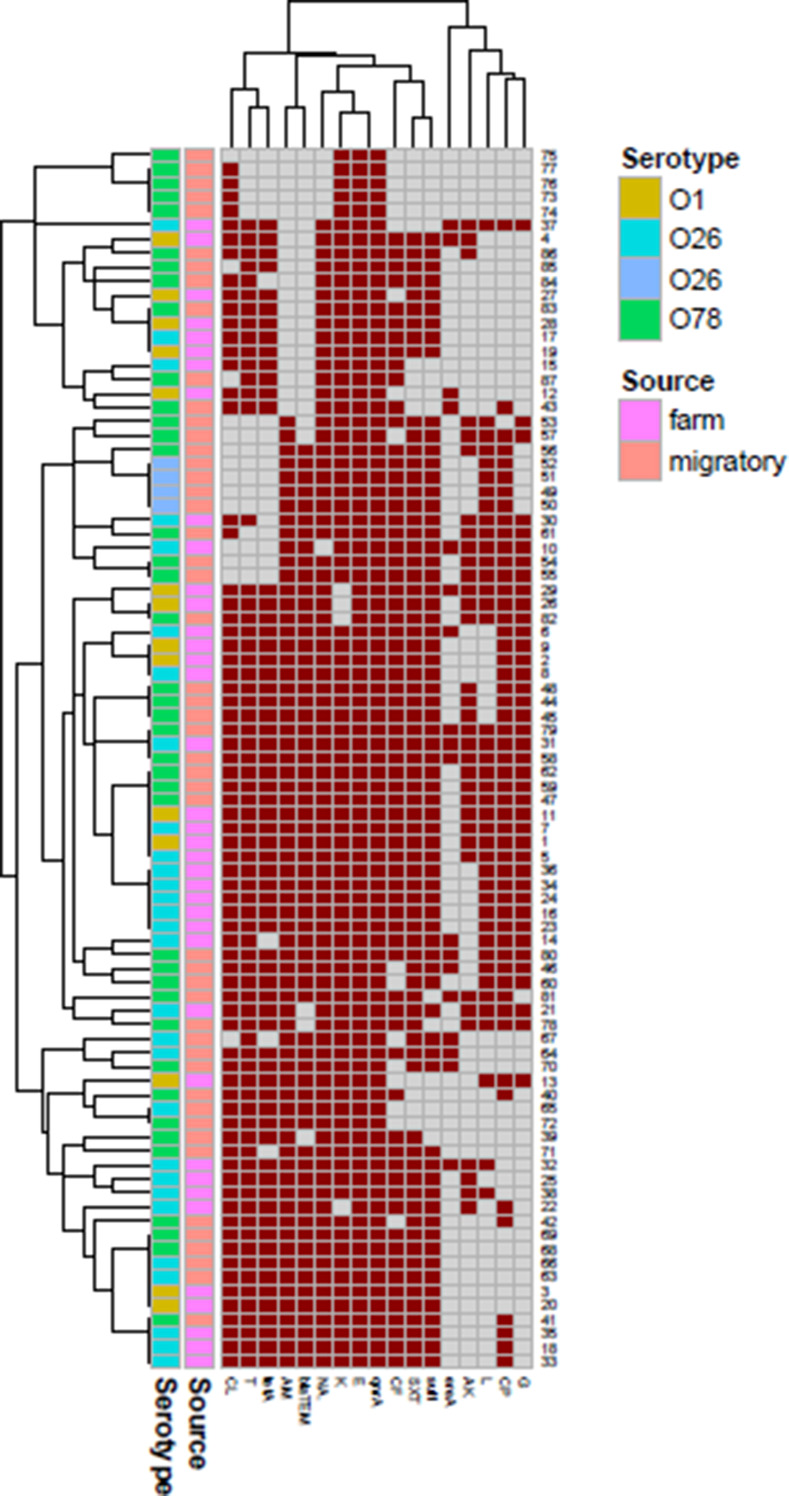
*E. coli* O1, O26 and O78 (the most important Pathogenic serotypes) which were isolated from migratory birds and broiler farms were selected and used to construct a heatmap. Clustering demonstrated predominant multidrug resistant strains were isolated from migratory birds and broiler farms

The heatmap (Fig. 10) showed that the most important pathogenic strains of *Salmonella* (Enteritidis, Typhimurium, Infantis and Kentucky) were isolated from migratory birds and nearly all the isolates were multidrug resistant with different MDR profiles. On the other hand, the heatmap (Fig. 11) showed that the most important pathogenic serotypes of *E. coli* including O1, O26 and O78 at which O78 were the predominant strain from migratory birds. All the isolates which were represented in the heatmap were multidrug resistant with different MDR profiles but, there were similarity between the strains of migratory birds and poultry chicken farms in these profiles for the same serotype.

## Discussion

*Salmonella* and *E. coli* are the most common pathogens affecting poultry, in the current study, the higher percentages of *Salmonella* isolation were reported from migratory birds in the year 2021 with 28% and from farms in 2019 and 2020 with 46.7% for each year. Some research studies that performed on migratory birds come in near similarity with the current study whereas *Salmonella* was isolated with 28.26% in Egypt [33] and 21.21% in Bangladesh [18].

The highest level of *Salmonella* from broiler farms was determined during 2019 and 2020 with 46.7% for each. Our findings were agreed with Al-baqir et al. [34] who isolated *Salmonella* from chickens in Egypt with 32.6%. Lower frequencies of *Salmonella* isolation were recorded by Leinyuy et al. [35] who isolated it with 7.14%, 13%, 11.50% and 18.78%, respectively. The serotyping of *Salmonella* in the current study revealed the detection of 3 predominant serotypes (*S.* Typhimurium, *S*. Kentucky and *S*. Enteritidis) from both migratory birds and farms which were common between migratory birds and farms in accordance with Al-baqir et al. [34]. On the other hand, we detected rare serovars such as.

*E. coli* was isolated from migratory birds with (38.7%, 2019), (26.7%, 2020), (34%, 2021), (40.7%, 2022) and (22.7%, 2023). Our findings concerned with *E. coli* detection in migratory birds agreed to some extent with Yuan et al. [36] who recovered *E. coli* with (34.7%) in China. In this study, *E. coli* was isolated from broiler farms with (78.3%, 2019), (83.3%, 2020), (76.7%, 2021), (86.7%, 2022) and (83.3%, 2023). A research study conducted by Tawakol and Younis [37] on migratory birds identified 4 serogroups (O125, O126, O158 and O86). From the migratory birds collected in Texas, Callaway et al. [38] isolated *E. coli* O157:H7 from 3.7% of samples. The observed results of *E. coli* recover from broiler farms in this study were in accordance with Ozaki et al. [39] who isolated O125, O1 and O6.

Another seven species of the *Enterobacteriacae* family were recorded in this study; *Citrobacter*, *Enterobacter*, *Klebsiella*, *Proteus*, *Providencia*, *Serratia*, *Hafnia* and all of them were isolated from migratory birds and farms except, *Hafnia* that was recorded only from migratory birds at 2019. The differentiation of these species showed the detection of *Citrobacter freundii*,* Enterobacter aerogenes*,* Enterobacter agglomerans*,* Enterobacter cloacae*,* Klebsiella pneumoniae*,* Proteus mirabilis*,* Proteus vulgaris*,* Providencia rettgeri* and *Serratia mascerans* (Supplementary Table 2). Our findings were nearly similar to Raza et al. [40] and Giacopello et al. [41]. The current results from broiler farms agreed with Leinyuy et al. [35] and Moawad et al. [17].

Regarding to the in vitro antimicrobial susceptibility testing, the majority of the recovered *Salmonella* and *E. coli* from both migratory birds and broiler farms exhibited higher resistance to nalidixic acid, ciprofloxacin, tetracycline, ampicillin, sulfamethoxazol, clindamycin and erythromycin and our results agreed Card et al. [7]. Misuse of antibiotics specialy quinolones as unspecific treatment, or in subtherapeutic doses for prophylaxis or as growth promoters in developing countries, enhance the generations of antibiotic resistance in bacteria [42].

Regarding to *Salmonella* from the farms, our findings agreed with Shalaby et al. [11] who recorded total resistance to ampicillin and erythromycin. A significant MDR of *Salmonella* was reported to ampicillin, gentamycin [34]. Majority of the isolated *Salmonella* from broiler farms and *E. coli* from both migratory birds and farms showed MDR to the tested antimicrobial agents. These findings agreed with Kamboh et al. [42] who distinguished MDR in *Salmonella* isolated from migratory birds and Tawakol and Younis [37] who reported MDR in *E. coli* isolates.

The detection of the resistance genes in this study (table, 6) showed that the highest prevalence of *sul1*, *tet*A (A), *bla*^TEM^ and *qnr*A genes were recorded in the resistant *Salmonella* and *E. coli* isolates from migratory birds and broiler farms. Our findings were nearly similar to Sharif et al. [3] who reported TEM gene in 100% of *S. enterica* which isolated from wild migratory birds. Regarding to the broiler farms, our findings nearly agreed with Alam et al. [43] who reported *tet*A and *bla*^*T*EM−1^ in *Salmonella* isolates with 97.14% and 82.85%, respectively. *bla*^TEM−1^, *tet* (A) and *sul1* genes were detected in *E. coli* isolates with prevalence of 36.4%, 80.5% and 6.8%, respectively from migratory wild birds in China [23]. Our results in harmony with that of Yapicier et al. [44] who reported *tet* (A) gene in 54.3% of *E. coli* isolated from wild birds in Turkey. Meanwhile, Islam et al. [6]. recorded *tet* (A), *qnr*A and *bla*^TEM^ with 100%, 35.71% and 95.24%, respectively.

ERIC-PCR and the heatmap indicated to the genetic similarity between the MDR *E. coli* strains that isolated from both migratory birds and poultry farms also, there was genetic similarity between MDR *Salmonella* strains that isolated from both migratory birds and poultry farms. These findings highlighted the potential role of migratory birds as vectors to disseminate MDR *Salmonella* and *E. coli* to poultry farms. This is necessitating to keep migratory birds under continuous antimicrobial surveillance programs with the application of biosecurity measures for the prevention of migratory birds from the entrance to poultry farms [45].

## Electronic supplementary material

Below is the link to the electronic supplementary material.

Supplementary Material 1

Supplementary Material 2

## Data Availability

The datasets used and/or analyzed during the current study were provided within the manuscript and supplementary information files.
