# Supplementary material for: Role of migratory birds as a risk factor for the transmission of multidrug resistant Salmonella enterica and Escherichia coli to broiler poultry farms and its surrounding environment
Source: BMC Res Notes. 2024 Oct 17;17:314. doi: 10.1186/s13104-024-06958-7 (PMC11487717; doi:10.1186/s13104-024-06958-7)
Supplement: Supplementary file 2 — Supplementary Material 2 [file 13104_2024_6958_MOESM2_ESM.docx]

**Supplementary table (1): Primers sequences, target genes, amplicon sizes and cycling conditions.**

| Target | Primers sequences | Amplified segment (bp) | Primary  denaturation | Amplification (35 cycles) | | | Final extension | Reference |
| --- | --- | --- | --- | --- | --- | --- | --- | --- |
|  |  |  |  | Secondary denaturation | Annealing | Extension |  |  |
| *TetA(A)* | GGTTCACTCGAACGACGTCA | 570 | 94˚C  5 min. | 94˚C  30 sec. | 50˚C  40 sec. | 72˚C  45 sec. | 72˚C  10 min. | [25] |
|  | CTGTCCGACAAGTTGCATGA |  |  |  |  |  |  |  |
| *qnrA* | ATTTCTCACGCCAGGATTTG | 516 | 94˚C  5 min. | 94˚C  30 sec. | 55˚C  40 sec. | 72˚C  45 sec. | 72˚C  10 min. | [26] |
|  | GATCGGCAAAGGTTAGGTCA |  |  |  |  |  |  |  |
| *ereA* | GCCGGTGCTCATGAACTTGAG | 420 | 94˚C  5 min. | 94˚C  30 sec. | 60˚C  40 sec. | 72˚C  45 sec. | 72˚C  10 min. | [27] |
|  | CGACTCTATTCGATCAGAGGC |  |  |  |  |  |  |  |
| *blaTEM* | ATCAGCAATAAACCAGC | 516 | 94˚C  5 min. | 94˚C  30 sec. | 54˚C  40 sec. | 72˚C  45 sec. | 72˚C  10 min. | [28] |
|  | CCCCGAAGAACGTTTTC |  |  |  |  |  |  |  |
| *Sul1* | CGG CGT GGG CTA CCT GAA CG | 433 | 94˚C  5 min. | 94˚C  30 sec. | 60˚C  40 sec. | 72˚C  45 sec. | 72˚C  10 min. | [29] |
|  | GCC GAT CGC GTG AAG TTC CG |  |  |  |  |  |  |  |
| ERIC | ATG TAA GCT CCT GGG GAT TCA C | Variable | 94˚C  5 min. | 94˚C  30 sec. | 52˚C  1 min. | 72˚C  1 min. | 72˚C  10 min. | [31] |
|  | AAG TAA GTG ACT GGG GTG AGC G |  |  |  |  |  |  |  |

**Supplementary table (2) number of the other differentiated members of *Enterobacteriaceae* isolated from migratory birds and farms**

| **Governorate** | **Year** | **Migratory birds** | | | **Broiler farms** | |
| --- | --- | --- | --- | --- | --- | --- |
|  |  | **Member** | **No.** | **Bird spp.** | **Member** | **No.** |
| Dakahlia | 2019 | *Citrobacter diversus* | 10 | Northern Northern shoveler- common teal | *Citrobacter diversus* | 1 |
|  |  | *Enterobacter aerogenes* | 10 | Northern Northern shoveler- common teal | *Citrobacter freundii* | 3 |
|  |  | *Enterobacter agglomerans* | 5 | common teal | *Enterobacter aerogenes* | 1 |
|  |  | *Hafnia species* | 5 | common teal | *Klebsiella pneumoniae* | 8 |
|  |  | *Klebsiella ozaenae* | 3 | common teal | *Proteus mirabilis* | 3 |
|  |  | *Proteus mirabilis* | 5 | Northern Northern shoveler- common teal | *Proteus vulgaris* | 4 |
|  |  | *Proteus vulgaris* | 5 | northern pintail |  |  |
| Damietta | 2019 | *Klebsiella pneumoniae* | 15 | Northern Northern shoveler- northern pintail | *Enterobacter aerogenes* | 3 |
|  |  | *Proteus mirabilis* | 15 | Northern Northern shoveler- common teal | *Klebsiella pneumoniae* | 7 |
|  |  | *Proteus vulgaris* | 10 | northern pintail | *Proteus mirabilis* | 6 |
|  |  | *Serratia liquefaciens* | 7 | common teal- northern pintail | *Providencia rettgeri* | 3 |
|  |  |  |  |  | *Serratia liquefaciens* | 1 |
| Port Said | 2019 | *Citrobacter freundii* | 9 | Northern Northern shoveler- northern pintail | *Enterobacter aerogenes* | 2 |
|  |  | *Enterobacter aerogenes* | 10 | common teal- northern pintail | *Enterobacter cloacae* | 7 |
|  |  | *Enterobacter cloacae* | 4 | northern pintail | *Klebsiella pneumoniae* | 8 |
|  |  | *Hafnia species* | 5 | common teal | *Proteus mirabilis* | 3 |
|  |  | *Proteus vulgaris* | 5 | common teal |  |  |
|  |  | *Providencia rettgeri* | 9 | Northern Northern shoveler- northern pintail |  |  |
| Dakahlia | 2020 | *Citrobacter freundii* | 5 | moorhen | *Citrobacter freundii* | 2 |
|  |  | *Klebsiella pneumoniae* | 25 | moorhen - common teal-Garganey | *Enterobacter cloacae* | 4 |
|  |  | *Proteus vulgaris* | 5 | common teal | *Klebsiella pneumoniae* | 9 |
|  |  | *Providencia rettgeri* | 5 | common teal | *Proteus mirabilis* | 3 |
|  |  |  |  |  | *Providencia rettgeri* | 2 |
| Damietta | 2020 | *Citrobacter freundii* | 5 | northern pintail | *Citrobacter freundii* | 1 |
|  |  | *Enterobacter aerogenes* | 5 | moorhen | *Klebsiella pneumoniae* | 6 |
|  |  | *Enterobacter cloacae* | 19 | Garganey | *Proteus mirabilis* | 9 |
|  |  | *Klebsiella pneumoniae* | 10 | common teal | *Providencia rettgeri* | 4 |
|  |  | *Providencia rettgeri* | 10 | moorhen |  |  |
|  |  | *Serratia mascerans* | 8 | moorhen |  |  |
| Port Said | 2020 | *Citrobacter freundii* | 15 | northern pintail- common teal | *Citrobacter freundii* | 6 |
|  |  | *Enterobacter aerogenes* | 5 | common teal | *Enterobacter cloacae* | 1 |
|  |  | *Enterobacter agglomerans* | 5 | northern pintail | *Klebsiella pneumoniae* | 11 |
|  |  | *Klebsiella pneumoniae* | 5 | moorhen | *Proteus mirabilis* | 2 |
|  |  | *Proteus mirabilis* | 5 | common teal |  |  |
|  |  | *Proteus vulgaris* | 5 | common teal |  |  |
|  |  | *Providencia rettgeri* | 5 | common teal |  |  |
| Dakahlia | 2021 | *Citrobacter diversus-Garganey* | 10 | common teal | *Citrobacter freundii* | 4 |
|  |  | *Enterobacter agglomerans* | 10 | Garganey | *Klebsiella pneumoniae* | 8 |
|  |  | *Enterobacter cloacae* | 8 | common teal | *Proteus mirabilis* | 5 |
|  |  | *Proteus mirabilis* | 8 | northern paintail | *Providencia rettgeri* | 3 |
|  |  | *Providencia rettgeri* | 5 | common teal |  |  |
|  |  | *Serratia liquefaciens* | 3 | Garganey |  |  |
| Damietta | 2021 | *Citrobacter diversus* | 10 | Shelduck-Garganey | *Citrobacter freundii* | 5 |
|  |  | *Klebsiella pneumoniae* | 15 | Shelduck- common teal- wigeon | *Enterobacter cloacae* | 4 |
|  |  | *Proteus mirabilis* | 9 | Shelduck | *Klebsiella pneumoniae* | 3 |
|  |  |  |  |  | *Proteus mirabilis* | 5 |
|  |  |  |  |  | *Providencia rettgeri* | 3 |
| Port Said | 2021 | *Citrobacter diversus-Garganey* | 15 | common teal- wigeon-northern paintail | *Enterobacter aerogenes* | 3 |
|  |  | *Klebsiella pneumoniae* | 20 | common teal- wigeon-northern paintail | *Klebsiella pneumoniae* | 8 |
|  |  | *Proteus mirabilis* | 14 | common teal- -northern paintail | *Proteus mirabilis* | 1 |
|  |  | *Providencia rettgeri* | 5 | northern paintail | *Proteus vulgaris* | 3 |
|  |  | *Serratia liquefaciens* | 5 | common teal | *Providencia rettgeri* | 4 |
|  |  |  |  |  | *Serratia liquefaciens* | 1 |
| Dakahlia | 2022 | *Citrobacter diversus* | 5 | Northern Northern shoveler | *Citrobacter freundii* | 1 |
|  |  | *Citrobacter freundii* | 8 | common teal- common pochard | *Enterobacter aerogenes* | 1 |
|  |  | *Klebsiella pneumoniae* | 15 | Northern Northern shoveler northern paintail | *Klebsiella pneumoniae* | 13 |
|  |  | *Proteus mirabilis* | 5 | common teal | *Proteus mirabilis* | 3 |
|  |  | *Proteus vulgaris* | 9 | northern paintail | *Serratia liquefaciens* | 2 |
|  |  | *Serratia liquefaciens* | 5 | Northern Northern shoveler |  |  |
| Damietta | 2022 | *Citrobacter diversus* | 10 | Northern Northern shoveler- common pochard | *Citrobacter freundii* | 3 |
|  |  | *Enterobacter aerogenes* | 5 | Northern Northern shoveler | *Klebsiella pneumoniae* | 8 |
|  |  | *Klebsiella pneumoniae* | 10 | Northern Northern shoveler | *Proteus mirabilis* | 6 |
|  |  | *Proteus mirabilis* | 15 | Northern Northern shoveler- northern paintail | *Providencia rettgeri* | 2 |
|  |  | *Proteus vulgaris* | 3 | Northern Northern shoveler | *Serratia liquefaciens* | 1 |
|  |  | *Providencia rettgeri* | 3 | northern paintail |  |  |
|  |  | *Serratia liquefaciens* | 10 | Northern Northern shoveler- northern paintail |  |  |
| Port Said | 2022 | *Citrobacter diversus* | 5 | common teal | *Citrobacter freundii* | 4 |
|  |  | *Citrobacter freundii* | 5 | common pochard | *Enterobacter cloacae* | 8 |
|  |  | *Enterobacter aerogenes* | 4 | common pochard | *Klebsiella pneumoniae* | 3 |
|  |  | *Klebsiella pneumoniae* | 3 | common pochard | *Proteus mirabilis* | 2 |
|  |  | *Providencia rettgeri* | 14 | common pochard- northern paintail | *Proteus vulgaris* | 2 |
| Dakahlia | 2023 | *Citrobacter freundii* | 15 | common teal -Northern Northern shoveler | *Citrobacter freundii* | 4 |
|  |  | *Enterobacter aerogenes* | 10 | common teal | *Providencia rettgeri* | 4 |
|  |  | *Providencia rettgeri* | 5 | Common pochard | *Klebsiella pneumoniae* | 5 |
|  |  | *Klebsiella pneumoniae* | 5 | Northern Northern shoveler | *Proteus vulgaris* | 1 |
|  |  | *Proteus mirabilis* | 5 | common teal | *Enterobacter aerogenes* | 6 |
|  |  | *Proteus vulgaris* | 5 | Northern pintail |  |  |
|  |  | *Serratia liquefaciens* | 5 | Common pochard |  |  |
| Damietta | 2023 | *Citrobacter freundii* | 10 | moorhen-Northern pintail | *Citrobacter freundii* | 2 |
|  |  | *Providencia rettgeri* | 9 | common teal | *Proteus mirabilis* | 2 |
|  |  | *Proteus mirabilis* | 5 | Northern pintail | *Enterobacter aerogenes* | 4 |
|  |  | *Serratia liquefaciens* | 5 | Northern pintail | *Enterobacter cloacae* | 4 |
|  |  | *Enterobacter agglomerans* | 5 | Northern Northern shoveler | *Serratia liquefaciens* | 3 |
|  |  |  |  |  | *Klebsiella pneumoniae* | 5 |
| Port Said | 2023 | *Citrobacter freundii* | 10 | moorhen-Northern Northern shoveler | *Citrobacter freundii* | 6 |
|  |  | *Enterobacter aerogenes* | 5 | common teal | *Klebsiella pneumoniae* | 4 |
|  |  | *Providencia rettgeri* | 10 | moorhen-Common pochard | *Proteus vulgaris* | 2 |
|  |  | *Klebsiella pneumoniae* | 15 | common teal -Northern Northern shoveler-Northern pintail | *Proteus mirabilis* | 4 |
|  |  | *Klebsiella pneumoniae* | 10 | moorhen | *Serratia liquefaciens* | 3 |
|  |  | *Proteus vulgaris* | 10 | Common pochard- common teal | *Providencia rettgeri* | 1 |

**Supplementary table (3) Antimicrobial resistance pattern profiles of the isolated Salmonellae from migratory birds and farms**

| **Year** | **Migratory birds** | | | | | **Broiler farms** | | | | |
| --- | --- | --- | --- | --- | --- | --- | --- | --- | --- | --- |
|  | **No.** | **Antimicrobial agents** | **no. of isolates** | **no. of resistance markers** | **MDRI** | **No.** | **Antimicrobial agents** | **no. of isolates** | **no. of resistance markers** | **MDRI** |
| 2019 | 1 | NA, AM, SXT , L, G | 1 | 5 | 0.42 | 1 | NA, AM, SXT, CL, T, CP, K, AK, CF, L, G | 1 | 11 | 0.92 |
| 2019 | 2 | NA, E, AM, SXT | 4 | 4 | 0.33 | 2 | NA, E, AM, SXT | 1 | 4 | 0.33 |
| 2019 | 3 | NA, E, AM, SXT , L, G | 1 | 6 | 0.5 | 3 | NA, E, AM, SXT, T, CP | 1 | 6 | 0.5 |
| 2019 | 4 | NA, E, AM, SXT, AK, CF | 1 | 6 | 0.5 | 4 | NA, E, AM, SXT, T, CP, K, AK | 1 | 8 | 0.67 |
| 2019 | 5 | NA, E, AM, SXT, CL, T | 2 | 6 | 0.5 | 5 | NA, E, AM, SXT, CL, T | 1 | 6 | 0.5 |
| 2019 | 6 | NA, E, AM, SXT, CL, T, CP, K, AK | 1 | 9 | 0.75 | 6 | NA, E, AM, SXT, CL, T, CF, L,G | 1 | 9 | 0.75 |
| 2019 | 7 | NA, E, AM, SXT, CL, T, CP, K, AK, CF | 2 | 10 | 0.83 | 7 | NA, E, AM, SXT, CL, T, CP | 3 | 7 | 0.58 |
| 2019 | 8 | NA, E, AM, SXT, T | 2 | 5 | 0.42 | 8 | NA, E, AM, SXT, CL, T, CP, G, L | 1 | 9 | 0.75 |
| 2019 | 9 | NA, E, AM, SXT, T, CP, K, AK, CF | 1 | 9 | 0.75 | 9 | NA, E, AM, SXT, CL, T, CP, K, AK | 1 | 9 | 0.75 |
| 2019 | 10 | NA, E, CF | 6 | 3 | 0.25 | 10 | NA, E, AM, SXT, CL, T, CP, K, AK, CF, G, L | 2 | 12 | 1 |
| 2019 | 11 | NA, E, CF, L, G | 2 | 4 | 0.33 | 11 | NA, E, AM, SXT, CL, T, CP, K, AK, CF, L | 1 | 11 | 0.92 |
| 2019 | 12 | NA, E, L, G | 1 | 3 | 0.25 | 12 | NA, E, AM, SXT, CL, T, CP, K, AK, CF, L, G | 1 | 12 | 1 |
| 2019 | 13 | NA, E, SXT, CL, T, CP, AK, CF, L | 1 | 9 | 0.75 | 13 | NA, E, AM, SXT, CL, T, CP, K, AK, CF, L,G | 3 | 12 | 1 |
| 2019 | 14 | NA, E, SXT, CL, T, CP, K, AK | 1 | 8 | 0.67 | 14 | NA, E, AM, SXT, CL, T, CP, K, AK, G | 2 | 10 | 0.83 |
| 2019 | 15 | NA, E, SXT, CL, T, CP, K, AK, CF, L | 1 | 10 | 0.83 | 15 | NA, E, AM, SXT, CL, T, CP, K, G | 2 | 9 | 0.75 |
| 2019 | 16 | NA, E, SXT, CL, T, CP, K, CF, L | 1 | 9 | 0.75 | 16 | NA, E, AM, SXT, CL, T, CP, L | 1 | 8 | 0.67 |
| 2019 | 17 | NA, E, SXT, T, CP, K, AK, CF, L | 1 | 9 | 0.75 | 17 | NA, E, AM, SXT, CL, T, L | 1 | 7 | 0.58 |
| 2019 | 18 | NA, L, G | 1 | 3 | 0.25 | 18 | NA, E, AM, T, CP, K, AK, CF, G, L | 1 | 10 | 0.83 |
| 2019 | 19 | NA, SXT | 1 | 2 | 0.17 | 19 | NA, E, AM, T, CP, K, AK, CF, L,G | 3 | 10 | 0.83 |
| 2019 | 20 | NA, SXT, CL, T | 1 | 4 | 0.33 |  |  |  |  |  |
| 2020 | 1 | NA, E | 9 | 2 | 0.17 | 1 | NA, E, AM, SXT, CL, T, AK, CF, L | 1 | 9 | 0.75 |
| 2020 | 2 | NA, E, AM, T, CP | 1 | 5 | 0.42 | 2 | NA, E, AM, SXT, CL, T, CP | 6 | 7 | 0.58 |
| 2020 | 3 | NA, E, AM, CL, T, CP, K, AK, CF | 1 | 10 | 0.83 | 3 | NA, E, AM, SXT, CL, T, CP, CF, L | 1 | 9 | 0.75 |
| 2020 | 4 | NA, E, AM, SXT | 1 | 4 | 0.33 | 4 | NA, E, AM, SXT, CL, T, CP, K, Ak | 1 | 9 | 0.75 |
| 2020 | 5 | NA, E, AM, SXT, CL CF, L | 1 | 7 | 0.58 | 5 | NA, E, AM, SXT, CL, T, CP, K, AK, CF | 2 | 10 | 0.83 |
| 2020 | 6 | NA, E, AM, SXT, CL, T | 5 | 6 | 0.5 | 6 | NA, E, AM, SXT, CL, T, CP, K, AK, CF, L | 14 | 11 | 0.92 |
| 2020 | 7 | NA, E, AM, SXT, CL, T, CF, L | 1 | 8 | 0.67 | 7 | NA, E, AM, SXT, CL, T, CP, K, AK, CF, L, G | 2 | 12 | 1 |
| 2020 | 8 | NA, E, AM, SXT, CL, T, CP | 2 | 7 | 0.58 | 8 | NA, E, AM, SXT, K, AK, CF, L | 1 | 8 | 0.67 |
| 2020 | 9 | NA, E, AM, SXT, CL, T, CP, AK, CF | 1 | 9 | 0.75 |  |  |  |  |  |
| 2020 | 10 | NA, E, AM, SXT, CL, T, CP, CF | 1 | 8 | 0.67 |  |  |  |  |  |
| 2020 | 11 | NA, E, AM, SXT, CL, T, CP, CF, L | 1 | 9 | 0.75 |  |  |  |  |  |
| 2020 | 12 | NA, E, AM, SXT, CL, T, CP, K, AK | 1 | 9 | 0.75 |  |  |  |  |  |
| 2020 | 13 | NA, E, AM, SXT, CL, T, CP, K, AK, CF | 7 | 10 | 0.83 |  |  |  |  |  |
| 2020 | 14 | NA, E, AM, SXT, CL, T, CP, K, AK, CF, L | 4 | 11 | 0.92 |  |  |  |  |  |
| 2020 | 15 | NA, E, AM, SXT, T, CP | 1 | 6 | 0.5 |  |  |  |  |  |
| 2021 | 1 | NA, AM, SXT | 2 | 3 | 0.25 | 1 | NA, E, AM, SXT, CL, L, G | 1 | 7 | 0.58 |
| 2021 | 2 | NA, AM, SXT, CL, T, CP, K, AK, CF, L | 1 | 10 | 0.83 | 2 | NA, E, AM, SXT, CL, CF, L, G | 1 | 8 | 0.67 |
| 2021 | 3 | NA, E | 10 | 2 | 0.17 | 3 | NA, E, AM, SXT, CL, T | 2 | 6 | 0.5 |
| 2021 | 4 | NA, E, AM | 2 | 3 | 0.25 | 4 | NA, E, AM, SXT, CL, T, L, G | 1 | 8 | 0.67 |
| 2021 | 5 | NA, E, AM, CL, T, CP, K | 1 | 7 | 0.58 | 5 | NA, E, AM, SXT, CL, T, CP | 1 | 8 | 0.67 |
| 2021 | 6 | NA, E, AM, SXT | 11 | 4 | 0.33 | 6 | NA, E, AM, SXT, CL, T, CP, K, AK, CF | 1 | 7 | 0.58 |
| 2021 | 7 | NA, E, AM, SXT, CL | 3 | 5 | 0.42 | 7 | NA, E, AM, SXT, CL, T, CP, K, AK, CF, L, G | 6 | 10 | 0.83 |
| 2021 | 8 | NA, E, AM, SXT, CL, T, CP, K, AK, CF, L | 1 | 11 | 0.92 | 8 | NA, E, AM, SXT, CL, T, CP, CF, G | 2 | 12 | 1 |
| 2021 | 9 | NA, E, AM, SXT, CL, T, CP, K | 2 | 8 | 0.67 | 9 | NA, E, AM, SXT, CL, T, CP, CF, L, G | 3 | 9 | 0.75 |
| 2021 | 10 | NA, E, AM, SXT, CL, T, CP, K, AK, CF | 1 | 10 | 0.83 | 10 | NA, E, AM, SXT, CL, T, CP, K, AK, CF | 1 | 10 | 0.83 |
| 2021 | 11 | NA, E, AM, SXT, CL, T, CP, K, AK, CF, L | 4 | 11 | 0.92 | 11 | NA, E, AM, SXT, CL, T, CP, K, AK, CF, L | 1 | 11 | 0.92 |
| 2021 | 12 | NA, E, AM, SXT, CL, T, CP, K, AK, CF, L, G | 1 | 12 | 1 |  |  |  |  |  |
| 2021 | 13 | NA, E, AM, SXT, CL, T, K, AK, CF, L | 1 | 10 | 0.83 |  |  |  |  |  |
| 2021 | 14 | NA, E, AM, SXT, T, CP, K | 1 | 7 | 0.58 |  |  |  |  |  |
| 2021 | 15 | NA, E, AM, SXT, T, CP, K, AK, CF, L | 1 | 10 | 0.83 |  |  |  |  |  |
| 2022 | 1 | NA, E, AM, SXT, CL, T | 8 | 6 | 0.5 | 1 | NA, E, AM, SXT | 1 | 4 | 0.33 |
| 2022 | 2 | NA, E, AM, SXT, CL, T, CP | 5 | 7 | 0.58 | 2 | NA, E, AM, SXT, CF, L, G | 1 | 7 | 0.58 |
| 2022 | 3 | NA, E, AM, SXT, CL, T, CP, K | 7 | 8 | 0.67 | 3 | NA, E, AM, SXT, CL, CP | 1 | 6 | 0.5 |
| 2022 | 4 | NA, E, AM, SXT, CL, T, CP, K, AK, CF, L, G | 5 | 12 | 1 | 4 | NA, E, AM, SXT, CL, G | 1 | 6 | 0.5 |
|  |  |  |  |  |  | 5 | NA, E, AM, SXT, CL, T | 1 | 6 | 0.5 |
|  |  |  |  |  |  | 6 | NA, E, AM, SXT, CL, T, CP | 5 | 7 | 0.58 |
|  |  |  |  |  |  | 7 | NA, E, AM, SXT, CL, T, CP, K, AK, CF, L, G | 7 | 12 | 1 |
|  |  |  |  |  |  | 8 | NA, E, AM, SXT, CL, T, G | 4 | 7 |  |
|  |  |  |  |  |  | 9 | NA, E, AM, SXT, K, AK, CF, L, G | 3 | 9 | 0.75 |
|  |  |  |  |  |  | 10 | NA, E, AM, SXT, T, CP | 1 | 6 | 0.5 |
|  |  |  |  |  |  | 11 | NA, SXT, CL, T, CP, K, AK, CF, L, G | 1 | 9 | 0.75 |
|  |  |  |  |  |  | 12 | NA, T, CP, K, AK, CF, L, G | 1 | 8 | 0.67 |
| 2023 | 1 | NA, AM | 2 | 2 | 0.17 | 1 | NA, E, AM, SXT | 2 | 4 | 0.33 |
|  | 2 | NA, E | 1 | 2 | 0.17 | 2 | NA, E, AM, SXT, CL, T, CP | 3 | 7 | 0.58 |
|  | 3 | NA, E, AM | 7 | 3 | 0.25 | 3 | NA, E, AM, SXT, CL, T, CP, CF, L, G | 2 | 10 | 0.83 |
|  | 4 | NA, E, AM, CL, T, CP, K, AK | 1 | 8 | 0.67 | 4 | NA, E, AM, SXT, CL, T, CP, K, AK, CF, L, G | 12 | 12 | 1 |
|  | 5 | NA, E, AM, SXT | 5 | 4 | 0.33 | 5 | NA, E, AM, SXT, CL, T, CP, L, G | 2 | 9 | 0.75 |
|  | 6 | NA, E, AM, SXT, CL | 1 | 5 | 0.42 | 6 | NA, E, AM, SXT, CL, T, G | 2 | 7 | 0.58 |
|  | 7 | NA, E, AM, SXT, CL, T | 1 | 6 | 0.5 | 7 | NA, E, AM, SXT, K, AK, CF, L, G | 2 | 9 | 0.75 |
|  | 8 | NA, E, AM, SXT, CL, T, CP | 3 | 7 | 0.58 | 8 | NA, E, AM, SXT, T, CP, CF, L, G | 1 | 9 | 0.75 |
|  | 9 | NA, E, AM, SXT, CL, T, CP, AK, CF | 1 | 9 | 0.75 |  |  |  |  |  |
|  | 10 | NA, E, AM, SXT, CL, T, CP, K, AK | 2 | 9 | 0.75 |  |  |  |  |  |
|  | 11 | NA, E, AM, SXT, CL, T, CP, K, AK, CF | 2 | 10 | 0.83 |  |  |  |  |  |
|  | 12 | NA, E, AM, SXT, CL, T, K, AK | 1 | 8 | 0.67 |  |  |  |  |  |
|  | 13 | NA, E, AM, SXT, K, AK | 1 | 6 | 0.5 |  |  |  |  |  |
|  | 14 | NA, E, AM, SXT, T | 3 | 5 | 0.42 |  |  |  |  |  |
|  | 15 | NA, E, AM, SXT, T, CP, K, AK, CF | 1 | 9 | 0.75 |  |  |  |  |  |
|  | 16 | NA, E, SXT, CL, T, CP, K, AK | 1 | 8 | 0.67 |  |  |  |  |  |

**Supplementary table (4) Antimicrobial resistance pattern profiles of the isolated *E. coil* from migratory birds and farms**

| **Year** | **Migratory birds** | | | | | **Broiler farms** | | | | |
| --- | --- | --- | --- | --- | --- | --- | --- | --- | --- | --- |
|  | **No.** | **Antimicrobial agents** | **no. of isolates** | **no. of resistance markers** | **MDRI** | **No.** | **Antimicrobial agents** | **no. of isolates** | **no. of resistance markers** | **MDRI** |
| 2019 | 1 | E, T, NA, CF, SXT, AM | 1 | 6 | 0.5 | 1 | E, CL, T, NA, CF, SXT | 1 | 6 | 0.5 |
| 2019 | 2 | E, T, NA, CF, SXT, AM, G, L, K, AK | 1 | 10 | 0.83 | 2 | E, CL, T, NA, CF, SXT, AM, L, K, AK | 2 | 10 | 0.83 |
| 2019 | 3 | E, CL, NA, CF, SXT, AM, CP, G, L, K, AK | 1 | 11 | 0.92 | 3 | E, CL, T, NA, CF, SXT, AM, CP | 4 | 8 | 0.67 |
| 2019 | 4 | E, CL, NA, CF, SXT, AM, G, L, K, AK | 1 | 10 | 0.83 | 4 | E, CL, T, NA, CF, SXT, AM, CP, G, L | 2 | 10 | 0.83 |
| 2019 | 5 | E, CL, T, NA, SXT, AM, CP | 1 | 7 | 0.58 | 5 | E, CL, T, NA, CF, SXT, AM, CP, G, L, K, AK | 5 | 12 | 1 |
| 2019 | 6 | E, CL, T, NA, AM, G, L, K, AK | 1 | 9 | 0.75 | 6 | E, CL, T, NA, CF, SXT, AM, AK | 1 | 8 | 0.67 |
| 2019 | 7 | E, CL, T, NA, CF, AM, CP | 1 | 7 | 0.58 | 7 | E, CL, T, NA, CF, SXT, AM, CP | 2 | 8 | 0.67 |
| 2019 | 8 | E, CL, T, NA, CF, CP | 1 | 6 | 0.5 | 8 | E, CL, T, NA, CF, SXT, AM, CP, G, L | 4 | 10 | 0.83 |
| 2019 | 9 | E, CL, T, NA, CF, CP, G, L, K, AK | 5 | 10 | 0.83 | 9 | E, CL, T, NA, CF, SXT, AM, CP, G, L, AK | 5 | 11 | 0.92 |
| 2019 | 10 | E, CL, T, NA, CF, SXT | 1 | 6 | 0.5 | 10 | E, CL, T, NA, CF, SXT, AM, CP, G, L, K | 1 | 11 | 0.92 |
| 2019 | 11 | E, CL, T, NA, CF, SXT, AM | 6 | 7 | 0.58 | 11 | E, CL, T, NA, CF, SXT, AM, CP, G, L, K, AK | 1 | 12 | 1 |
| 2019 | 12 | E, CL, T, NA, CF, SXT, AM, AK | 5 | 8 | 0.67 | 12 | E, CL, T, NA, CF, SXT, AM, K, AK | 1 | 9 | 0.75 |
| 2019 | 13 | E, CL, T, NA, CF, SXT, AM, CP | 1 | 8 | 0.67 | 13 | E, CL, T, NA, CF, SXT, L, K, AK | 1 | 9 | 0.75 |
| 2019 | 14 | E, CL, T, NA, CF, SXT, AM, CP, G, L, K, AK | 6 | 12 | 1 | 14 | E, CL, T, NA, CP, G, L, K, AK | 1 | 9 | 0.75 |
| 2019 | 15 | E, CL, T, NA, CF, SXT, AM, G, L, K, AK | 1 | 11 | 0.92 | 15 | E, CL, T, NA, SXT, AM, CP, AK | 1 | 8 | 0.67 |
| 2019 | 16 | E, CL, T, NA, CF, SXT, AM, K | 1 | 8 | 0.67 | 16 | E, CL, T, NA, SXT, AM, CP, G, L | 1 | 9 | 0.75 |
| 2019 | 17 | E, CL, T, NA, CF, SXT, AM, K, AK | 2 | 9 | 0.75 | 17 | E, CL, T, NA, SXT, AM, CP, G, L, AK | 1 | 10 | 0.83 |
| 2019 | 18 | E, CL, T, NA, SXT, AM, CP, G, L | 2 | 9 | 0.75 | 18 | E, CL, T, NA, SXT, CP, G, L, K | 3 | 9 | 0.75 |
| 2019 | 19 | E, CL, T, NA, SXT, AM, CP, G, L, AK | 3 | 10 | 0.83 | 19 | E, NA, CF, SXT, AM | 3 | 5 | 0.42 |
| 2019 | 20 | E, CL, T, NA, SXT, AM, G, L, K, AK | 1 | 10 | 0.83 | 20 | E, NA, CF, SXT, AM, CP | 1 | 6 | 0.5 |
| 2019 | 21 | E, NA, CF, SXT, AM, CP, G, L, K, AK | 2 | 10 | 0.83 | 21 | E, NA, CF, SXT, AM, CP, G, L, K, AK | 1 | 10 | 0.83 |
| 2019 | 22 | E, NA, CF, SXT, AM, CP, L | 4 | 7 | 0.58 | 22 | E, T, NA, CF, SXT, AM | 2 | 6 | 0.5 |
| 2019 | 23 | E, NA, CF, SXT, AM, CP, L, K, AK | 1 | 7 | 0.58 | 23 | E, T, NA, CF, SXT, AM, CP, G, L | 1 | 9 | 0.75 |
| 2019 | 24 | E, NA, CF, SXT, AM, G, L, K, AK | 1 | 9 | 0.75 | 24 | T, NA, CF, SXT, AM, CP, G, L, K, AK | 1 | 10 | 0.83 |
| 2019 | 25 | E, NA, SXT, AM, CP, G, L, AK | 1 | 8 | 0.67 | 25 | T, NA, CF, SXT, AM, CP, G, L, AK | 1 | 9 | 0.75 |
| 2019 | 26 | E, NA, SXT, AM, CP, G, L, K, AK | 1 | 9 | 0.75 |  |  |  |  |  |
| 2019 | 27 | E, T, NA, CF, SXT, L, K, AK | 4 | 8 | 0.67 |  |  |  |  |  |
| 2019 | 28 | E, T, NA, SXT, AM, CP, G, L | 1 | 8 | 0.67 |  |  |  |  |  |
| 2019 | 29 | CL, T, NA, CF, SXT, AM, CP, G, L, K, AK | 1 | 11 | 0.92 |  |  |  |  |  |
| 2020 | 1 | E | 9 | 1 | 0.08 | 1 | E, T, NA, CF, SXT, AM, CP, G | 1 | 8 | 0.67 |
| 2020 | 2 | E, CL, NA | 1 | 3 | 0.25 | 2 | E, CL, T, NA, AM | 1 | 5 | 0.42 |
| 2020 | 3 | E, CL, T, NA | 2 | 4 | 0.33 | 3 | E, CL, T, NA, AM, CP, G | 1 | 7 | 0.58 |
| 2020 | 4 | E, CL, T, NA, AM | 2 | 5 | 0.42 | 4 | E, CL, T, NA, AM, CP, G, L, K, AK | 1 | 10 | 0.83 |
| 2020 | 5 | E, CL, T, NA, CF, SXT | 4 | 6 | 0.5 | 5 | E, CL, T, NA, CF, G, L, K, AK | 3 | 9 | 0.75 |
| 2020 | 6 | E, CL, T, NA, CF, SXT, AM | 9 | 7 | 0.58 | 6 | E, CL, T, NA, CF, SXT | 3 | 6 | 0.5 |
| 2020 | 7 | E, CL, T, NA, CF, SXT, AM, CP, G | 1 | 9 | 0.75 | 7 | E, CL, T, NA, CF, SXT, G, L | 1 | 8 | 0.67 |
| 2020 | 8 | E, CL, T, NA, CF, SXT, AM, CP, G, L | 1 | 10 | 0.83 | 8 | E, CL, T, NA, CF, SXT, AM | 6 | 7 | 0.58 |
| 2020 | 9 | E, CL, T, NA, CF, SXT, AM, CP, G, L, K | 3 | 11 | 0.92 | 9 | E, CL, T, NA, CF, SXT, AM, CP, AK | 1 | 9 | 0.75 |
| 2020 | 10 | E, CL, T, NA, CF, SXT, AM, CP, G, L, K, AK | 3 | 12 | 1 | 10 | E, CL, T, NA, CF, SXT, AM, CP, G | 6 | 9 | 0.75 |
| 2020 | 11 | E, CL, T, NA, CF, SXT, AM, CP, L, K | 1 | 10 | 0.83 | 11 | E, CL, T, NA, CF, SXT, AM, CP, G, L | 3 | 10 | 0.83 |
| 2020 | 12 | E, CL, T, NA, CF, SXT, L, K | 1 | 8 | 0.67 | 12 | E, CL, T, NA, CF, SXT, AM, CP, G, L, AK | 2 | 11 | 0.92 |
| 2020 | 13 | E, CL, T, NA, SXT, AM | 1 | 6 | 0.5 | 13 | E, CL, T, NA, CF, SXT, AM, CP, G, L, K | 3 | 11 | 0.92 |
| 2020 | 14 | E, T, NA | 1 | 3 | 0.25 | 14 | E, CL, T, NA, CF, SXT, AM, CP, G, L, K, AK | 11 | 12 | 1 |
| 2020 | 15 | E, T, NA, SXT, AM | 1 | 5 | 0.42 | 15 | E, CL, T, NA, CF, SXT, AM, CP, K | 1 | 9 | 0.75 |
|  |  |  |  |  |  | 16 | E, CL, T, NA, CF, SXT, AM, K, Ak | 1 | 9 | 0.75 |
|  |  |  |  |  |  | 17 | E, CL, T, NA, CF, SXT, L, K | 1 | 8 | 0.67 |
|  |  |  |  |  |  | 18 | E, CL, T, NA, SXT | 1 | 5 | 0.42 |
|  |  |  |  |  |  | 19 | E, CL, T, NA, SXT, AM | 1 | 6 | 0.5 |
|  |  |  |  |  |  | 20 | E, CL, T, NA, SXT, AM, CP, G, L, K, AK | 1 | 11 | 0.92 |
|  |  |  |  |  |  | 21 | E, T, NA, SXT, AM, CP, G, L | 1 | 8 | 0.67 |
| 2021 | 1 | E | 1 | 1 | 0.08 | 1 | E, CL, T, NA, CF | 4 | 5 | 0.42 |
| 2021 | 2 | E, CL | 5 | 2 | 0.17 | 2 | E, CL, T, NA, CF, SXT | 6 | 6 | 0.5 |
| 2021 | 3 | E, CL, NA, CF, SXT, AM, CP, L, K | 1 | 9 | 0.75 | 3 | E, CL, T, NA, CF, SXT, AM | 6 | 7 | 0.58 |
| 2021 | 4 | E, CL, T | 8 | 3 | 0.25 | 4 | E, CL, T, NA, CF, SXT, AM, CP | 3 | 8 | 0.67 |
| 2021 | 5 | E, CL, T, NA, CF, AM | 1 | 6 | 0.5 | 5 | E, CL, T, NA, CF, SXT, AM, CP, G | 5 | 9 | 0.75 |
| 2021 | 6 | E, CL, T, NA, CF, AM, CP | 1 | 7 | 0.58 | 6 | E, CL, T, NA, CF, SXT, AM, CP, G, L | 9 | 10 | 0.83 |
| 2021 | 7 | E, CL, T, NA, CF, SXT | 7 | 6 | 0.5 | 7 | E, CL, T, NA, CF, SXT, AM, CP, G, L, K | 3 | 11 | 0.92 |
| 2021 | 8 | E, CL, T, NA, CF, SXT, AM | 7 | 7 | 0.58 | 8 | E, CL, T, NA, CF, SXT, AM, CP, G, L, K, AK | 9 | 12 | 1 |
| 2021 | 9 | E, CL, T, NA, CF, SXT, AM, CP | 2 | 8 | 0.67 | 9 | E, CL, T, NA, CF, SXT, AM, G, L | 1 | 9 | 0.75 |
| 2021 | 10 | E, CL, T, NA, CF, SXT, AM, CP, G, L, AK | 1 | 10 | 0.83 | 10 | E, CL, T, NA, CF | 4 | 5 | 0.42 |
| 2021 | 11 | E, CL, T, NA, CF, SXT, AM, CP, G, L, K | 7 | 11 | 0.92 |  |  |  |  |  |
| 2021 | 12 | E, CL, T, NA, CF, SXT, AM, CP, G, L, K, AK | 4 | 12 | 1 |  |  |  |  |  |
| 2021 | 13 | E, CL, T, NA, CF, SXT, AM, CP, L, K, AK | 1 | 11 | 0.92 |  |  |  |  |  |
| 2021 | 14 | E, CL, T, NA, SXT | 1 | 5 | 0.42 |  |  |  |  |  |
| 2021 | 15 | E, CL, T, NA, SXT, AM | 1 | 6 | 0.5 |  |  |  |  |  |
| 2021 | 16 | E, CL, T, NA, SXT, AM, CP | 1 | 7 | 0.58 |  |  |  |  |  |
| 2021 | 17 | E, T | 1 | 2 | 0.17 |  |  |  |  |  |
| 2021 | 18 | E, T, NA, CF, SXT, AM, CP, G, L, K | 1 | 10 | 0.83 |  |  |  |  |  |
| 2022 | 1 | E | 5 | 1 | 0.08 | 1 | CL, T, NA, CF, SXT | 1 | 5 | 0.42 |
| 2022 | 2 | E, CL | 9 | 2 | 0.17 | 2 | E, CL, CF, SXT, AM, CP, G, L, K | 1 | 9 | 0.75 |
| 2022 | 3 | E, CL, T, NA | 8 | 4 | 0.33 | 3 | E, CL, T, CF, SXT, G | 1 | 6 | 0.5 |
| 2022 | 4 | E, CL, T, NA, CF | 5 | 5 | 0.42 | 4 | E, CL, T, CF, SXT, G, L, K, AK | 1 | 9 | 0.75 |
| 2022 | 5 | E, CL, T, NA, CF, SXT | 10 | 6 | 0.5 | 5 | E, CL, T, NA | 1 | 4 | 0.33 |
| 2022 | 6 | E, CL, T, NA, CF, SXT, AM, CP, G, L, K, AK | 9 | 12 | 1 | 6 | E, CL, T, NA, CF, L, K, AK | 1 | 8 | 0.67 |
| 2022 | 7 | E, CL, T, NA, CF, SXT, AM, CP, G, L | 5 | 10 | 0.83 | 7 | E, CL, T, NA, CF, SXT | 4 | 6 | 0.5 |
| 2022 | 8 | E, CL, T, NA, CF, SXT, AM, CP, G, L, K | 5 | 11 | 0.92 | 8 | E, CL, T, NA, CF, SXT, AM | 4 | 7 | 0.58 |
| 2022 | 9 | E, CL, T, NA, SXT, AM | 5 | 6 | 0.5 | 9 | E, CL, T, NA, CF, SXT, AM, CP | 2 | 8 | 0.67 |
|  |  |  |  |  |  | 10 | E, CL, T, NA, CF, SXT, AM, CP, G | 1 | 9 | 0.75 |
|  |  |  |  |  |  | 11 | E, CL, T, NA, CF, SXT, AM, CP, G, AK | 4 | 10 | 0.83 |
|  |  |  |  |  |  | 12 | E, CL, T, NA, CF, SXT, AM, CP, G, L | 1 | 9 | 0.75 |
|  |  |  |  |  |  | 13 | E, CL, T, NA, CF, SXT, AM, CP, G, L, K | 1 | 11 | 0.92 |
|  |  |  |  |  |  | 14 | E, CL, T, NA, CF, SXT, AM, CP, G, L, K, AK | 10 | 12 | 1 |
|  |  |  |  |  |  | 15 | E, CL, T, NA, AM, CP, G, L, K | 2 | 9 | 0.75 |
|  |  |  |  |  |  | 16 | E, CL, T, NA, CF, SXT, AM, L, K, AK | 6 | 10 | 0.83 |
|  |  |  |  |  |  | 17 | E, CL, T, NA, CF, SXT, CP, G, L, K, AK | 1 | 11 | 0.92 |
|  |  |  |  |  |  | 18 | E, CL, T, NA, CF, SXT, CP, L, K, AK | 1 | 10 | 0.83 |
|  |  |  |  |  |  | 19 | E, CL, T, NA, CF, SXT, G, L, K | 4 | 9 | 0.75 |
|  |  |  |  |  |  | 20 | E, CL, T, NA, CF, SXT, G, L, K, AK | 3 | 10 | 0.83 |
|  |  |  |  |  |  | 21 | NA, CF, SXT, AM, L, K, AK | 1 | 7 | 0.58 |
|  |  |  |  |  |  | 22 | T, NA, CF, SXT, AM, CP, G, L, K, AK | 1 | 10 | 0.83 |
| 2023 | 1 | E, CL | 9 | 2 | 0.17 | 1 | E, CF, SXT, AM, CP, G, L, K, AK | 1 | 9 | 0.75 |
| 2023 | 2 | E, CL, T, NA | 5 | 4 | 0.33 | 2 | E, AM, CP, G, L, K, AK | 2 | 7 | 0.58 |
| 2023 | 3 | E, CL, T, NA, CF, SXT | 8 | 6 | 0.5 | 3 | E, CL, T, NA, CF | 1 | 5 | 0.42 |
| 2023 | 4 | E, CL, T, NA, CF, SXT, AM, CP, G, L | 5 | 10 | 0.83 | 4 | E, CL, T, NA, CF, SXT | 1 | 6 | 0.5 |
| 2023 | 5 | E, CL, T, NA, CF, SXT, AM, G, L | 4 | 9 | 0.75 | 5 | E, CL, T, NA, CF, SXT, AM | 10 | 8 | 0.67 |
| 2023 | 6 | E, CL, T, NA, CF, SXT, AM, L | 1 | 8 | 0.67 | 6 | E, CL, T, NA, CF, SXT, AM, CP | 3 | 8 | 0.67 |
| 2023 | 7 | E, T, NA, CF | 1 | 4 | 0.33 | 7 | E, CL, T, NA, CF, SXT, AM, CP, G | 7 | 7 | 0.58 |
| 2023 | 8 | E, T, NA, CF, SXT | 1 | 5 | 0.42 | 8 | E, CL, T, NA, CF, SXT, AM, CP, G, L, K, AK | 10 | 12 | 1 |
|  |  |  |  |  |  | 9 | E, CL, T, NA, CF, SXT, AM, K, AK | 2 | 9 | 0.75 |
|  |  |  |  |  |  | 10 | E, CL, T, NA, CF, SXT, K, AK | 8 | 8 | 0.67 |
|  |  |  |  |  |  | 11 | E, CL, T, NA, G, L, K, AK | 1 | 8 | 0.67 |
|  |  |  |  |  |  | 12 | E, CL, T, NA, SXT, AM, AK | 1 | 7 | 0.58 |
|  |  |  |  |  |  | 13 | E, CL, T, SXT, AM, CP, G, L, K, AK | 1 | 10 | 0.83 |
|  |  |  |  |  |  | 14 | E, NA, CF, SXT, AM, CP, G, L, K, AK | 1 | 10 | 0.83 |
|  |  |  |  |  |  | 15 | T, NA, CF, SXT, AM, CP, G, L, K, AK | 1 | 10 | 0.83 |
